# Novel Potent Hypoglycemic Compounds from *Euonymus laxiflorus* Champ. and Their Effect on Reducing Plasma Glucose in an ICR Mouse Model

**DOI:** 10.3390/molecules23081928

**Published:** 2018-08-02

**Authors:** Van Bon Nguyen, San-Lang Wang, Thi Hanh Nguyen, Minh Trung Nguyen, Chien Thang Doan, Thi Ngoc Tran, Zhi-Hu Lin, Quang Vinh Nguyen, Yao-Haur Kuo, Anh Dzung Nguyen

**Affiliations:** 1 Institute of Research and Development, Duy Tan University, Da Nang 550000, Vietnam; 2 Department of Science and Technology, Tay Nguyen University, Buon Ma Thuot 630000, Vietnam; nguyenhanh2208.tn@gmail.com (T.H.N.); nguyenminhtrung2389@gmail.com (M.T.N.); doanthng@gmail.com (C.T.D.); tranngoctnu@gmail.com (T.N.T.); 3 Department of Chemistry, Tamkang University, New Taipei City 25137, Taiwan; 4 Life Science Development Center, Tamkang University, New Taipei City 25137, Taiwan; 5 Division of Chinese Materia Medica Development, National Research Institute of Chinese Medicine, Taipei 11221, Taiwan; tiger77749@gmail.com; 6 Institute of Biotechnology and Environment, Tay Nguyen University, Buon Ma Thuot 630000, Vietnam; vinh12b@yahoo.com; 7 Graduate Institute of Integrated Medicine, College of Chinese Medicine, China Medical University, Taichung 40402, Taiwan

**Keywords:** *Euonymus laxiflorus* Champ., diabetes, α-glucosidase inhibitors, plasma glucose, condensed tannins, natural products

## Abstract

α-Glucosidase inhibitors (aGIs) have been used as an effective therapy for type-2 diabetes, which remains a global health issue. The aim of this study was to achieve bioactivity-guided isolation, identification and evaluation of hypoglycemic compounds from *Euonymus laxiflorus* Champ. trunk bark (ELCTB). Eleven active compounds were isolated and identified as walterolactone A/B β-d-pyranoglucoside (**1**), 1-β-d-glucopyranosyloxy-3,5-dimethoxy-4-hydroxybenzene (**9**), (−)-gallocatechin (**10**), schweinfurthinol 9-*O*-β-d-pyranoglucoside (**11**), 1-*O*-(3-methyl)-butenoyl-myo-inositol (**12**), leonuriside (**14**), (+)-catechin (**19**), methyl galloate (**20**), (−)-catechin (**23**), and condensed tannins (**5** and **18**). Of these 11, novel 4 compounds (**1**, **11**, **12**, and **14**) were found as new α-glucosidase inhibitors. Notably, in vitro results indicated that compounds **1**, **5**, **10**–**12**, **18**, and **19** showed potent activity (IC_50_ = 0.076−31 µg/mL), and their activities were at a higher level than that of acarbose, a commercial inhibitor (IC_50_ = 1345 µg/mL). In animal tests, the major inhibitor, condensed tannin (**18**), demonstrated significant reduction of plasma glucose in mice with no symptoms of diarrhea at the dose of 100 mg/kg bw. The results suggest that *Euonymus laxiflorus* Champ. is a rich source of bioactive compounds for development as health food or drugs with potent hypoglycemic effect. The results of this study also enriched the current novel biological activities of constituents from *Euonymus laxiflorus* species.

## 1. Introduction

Natural bioactive products are of great interest due to their beneficial use as health foods or drugs to manage significant numbers of diseases including type-2 diabetes (T2D), a serious current global health issue [1,2]. Several therapies, including the use of α-glucosidase inhibitors (aGIs), have been applied for T2D management [3]. To date, some commercial aGIs have become available for T2D treatment, such as acarbose, miglitol, and voglibose. However, the use of these commercial drugs was reported to cause several side effects, such as diarrhea, flatulence, and abdominal discomfort [4]. Thus, the investigation of natural sources of aGIs for safe use is required.

aGIs could be obtained from various sources, such as herbal extracts [4,5,6,7], biosynthesis via microbial fermentation [2,8,9,10,11,12], or chemical synthesis [13,14]. Herbals were suggested as rich natural sources of aGIs, which may be useful in providing therapy for T2D [4,5]; as such, the bioassay-guided isolation and identification of active aGIs from potent antidiabetic herbals have proven valuable in research.

*Euonymus laxiflorus* Champ. is distributed in some Asian countries, including China, Vietnam, Cambodia, India, and Myanmar [15]. This herbal species was evaluated as the most potent source of aGIs and α-amylase inhibitors (aAIs) among various medicinal herbals collected in the central highlands of Vietnam in our previous studies [4,16]. The significant effect on the reduction of plasma glucose in diabetic rats of the methanolic extract of *Euonymus laxiflorus* Champ. trunk bark (ELCTB) was also previously recorded [17]. Recently, some compounds possessing α-amylase inhibitory activity were isolated and identified from the methanolic extract of the trunk bark of *Euonymus laxiflorus* Champ. [18]; as well, several compounds showing anti-nitro oxide activity were isolated from its leaves [19]. However, no compounds showing α-glucosidase inhibitory property were reported to be isolated and identified from this herbal species.

As part of our ongoing objective to develop *Euonymus laxiflorus* Champ. as a health food and drug with potent hypoglycemic function, the methanolic extract of ELCTB was conducted to isolate and identify active α-glucosidase inhibitors. The major and most active inhibitor was also tested for its effect on reducing blood glucose in mice.

## 2. Results and Discussion

### 2.1. Reclamation of Euonymus laxiflorus Champ. Extracts as a Potent Source of Natural aGIs

The methanol extract of *Euonymus laxiflorus* Champ. trunk bark (ELCTB) was newly found to be the most potent source of aGIs among 26 samples of indigenous medicinal plants collected in Dak Lak Province, Vietnam [4], showing effective inhibition against various α-glucosidases from rat (IC_50_ = 360 µg/mL), *S. cerevisiae* (IC_50_ = 1.32 µg/mL), and *B. stearothermophilus* (IC_50_ = 5.15 µg/mL).

In the comparison, this herbal extract demonstrated comparable or much higher α-glucosidase inhibitory activity compared to those of extracts from other reported herbals also collected in the central highlands of Vietnam due to its smallest IC_50_ values against α-glucosidases from rat (Table 1).

The methanol extract of this herbal also possesses significant effect on reducing plasma glucose in diabetic rats [17]. Thus, it was used for bioactivity-guided isolation of active hypoglycemic compounds via several columns, such as Diaion, Octadecylsilane, and preparative HPLC columns in the current report.

### 2.2. Purification of Active aGIs from MeOH Extract of ELCTB

#### 2.2.1. Separation, Subfractionation and Isolation of Compounds from ELCTB Extract

The methanol extract of *Euonymus laxiflorus* Champ. trunk bark (ELCTB) was first separated via a Diaion column to obtain five fractions. The crude sample and its fractions were tested for their inhibition against yeast α-glucosidase, and the results are presented in Table 2. The fractions **ELCTB-2** (eluted with 40% MeOH) and **ELCTB-3** (eluted with 70% MeOH) demonstrated the highest activity with low IC_50_ and great inhibition (%) values of 2.80 μg/mL, 99%, and 3.50 μg/mL, 98%, respectively. Thus, they were chosen for further separation.

**Figure 1:**
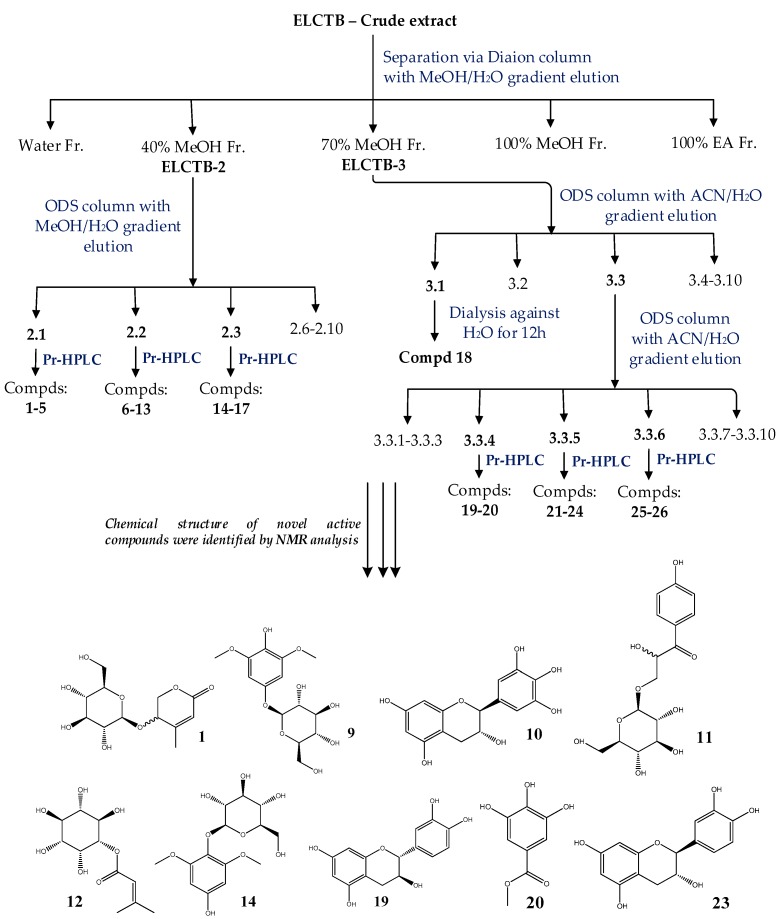
Flow chart on the purification and identification of active compounds from ELCTB extract. ACN: acetonitrile; ODS: octadecylsilane; Compds: compounds; Pr-HPLC: preparative high-performance liquid chromatography. (**1**): Walterolactone A/B β-d-pyranoglucoside; (**9**): 1-β-d-glucopyranosyloxy-3,5-dimethoxy-4-hydroxybenzene; (**10**): (−)-gallocatechin; (**11**): schweinfurthinol 9-*O*-β-d-pyranoglucoside; (**12**): 1-*O*-(3-methyl)-butenoyl-myo-inositol; (**14**): leonuriside; (**19**): (+)-catechin; (**20**): methyl galloate; (**23**): (−)-catechin.

Fractions **ELCTB-2** and **ELCTB-3** were loaded onto ODS columns and eluted with MeOH and ACN in H_2_O, respectively, to obtain 10 subfractions from each fraction, and their α-glucosidase inhibitory activity was tested. As shown in Table 2, subfraction **ELCTB-2.1** showed good activity (IC_50_ = 6.20 µg/mL, max inhibition = 97%). Similarly, subfraction **ELCTB-3.1** demonstrated the highest activity (IC_50_ = 1.12 µg/mL, max inhibition = 100%) among the 10 subfractions separated from **ELCTB-3**. Thus, these 2 potent subfractions were chosen for the isolation of active compounds. The other three fractions: **ELCTB-2.2**, **ELCTB-2.3**, and **ELCTB-3.3** were also considered for further purification due to their good TLC separation profiles. From these five subfractions (**ELCTB-2.1**, **ELCTB-2.2**, **ELCTB-2.3**, **ELCTB-3.1**, and **ELCTB-3.3**), a total of 26 compounds were isolated by utilizing preparative HPLC. The purification process is summarized in Figure 1.

#### 2.2.2. Evaluation and Identification of Active aGIs

**Figure 2:**
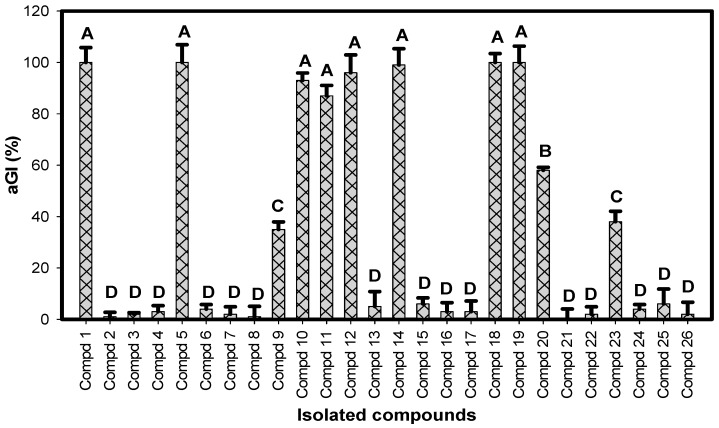
Evaluation of α-glucosidase inhibition (aGI (%)) of isolated compounds. All compounds were tested at a concentration of 250 µg/mL. Results are means ± SD of multi tests (*n* = 3). Coefficient of variation (%) = 21.72221. Mean values with the different letters are significantly different based on Duncan’s Multiple Range Test (alpha = 0.01).

The 26 isolated compounds were primarily tested for their inhibition against α-glucosidase at the same concentration of 250 µg/mL; the α-glucosidase inhibition, aGI (%), is presented in Figure 2. Eight compounds: **1**, **5**, **10**, **11**, **12**, **14**, **18**, and **19**, demonstrated potent activity (90–100%, ranked at level A), and three compounds: **9**, **20**, and **23**, showed activity of 38–60% (ranked at level B–C based on Duncan’s Multiple Range Test (alpha = 0.01)).

The chemical structures of the active compounds (**1**, **5**, **9**, **10**, **11**, **12**, **14**, **18**, **19**, **20**, and **23**) were identified via analysis of their NMR data, including ^1^H-MNR, ^13^C-NMR, COSY, HSQC, and HMBC coupled with the comparison of those of reported compounds. These active compounds were identified as walterolactone A/B β-d-pyranoglucoside (**1**) [18]; 1-β-d-glucopyranosyloxy-3,5-dimethoxy-4-hydroxybenzene (**9**) [20]; (−)-gallocatechin (**10**) [21]; schweinfurthinol 9-*O*-β-d-pyranoglucoside (**11**) [18]; 1-*O*-(3-methyl)-butenoyl-myo-inositol (**12**) [18]; leonuriside (**14**) [22,23]; (+)-catechin (**19**) [21]; methyl galloate (**20**) [24]; (−)-catechin (**23**) [21]; and condensed tannin (**5** and **18**) [18]. The NMR data of all these identified compounds are presented in the materials and methods section.

Compounds **5** & **18** were confirmed as condensed tannins by using total phenolic determination, biological assays [25], and comparison of their ^13^C-NMR data with those of reported condensed tannins [18,26,27]. These 2 compounds were pre-confirmed as tannins since they contained ≥94% total phenolic acid and also exhibited tested biological activities, including antioxidant activity (≥96%), α-amylase inhibition (100%), and protease inhibition (≥95%) at their tested concentration of 1 mg/mL. In addition, the ^13^C-NMR data on these compounds (materials and methods section) were similar to those of condensed tannin ELC3.1-d isolated and identified from the same herbal species [18], and also closely similar to those of other reported condensed tannins, including persimmon tannin [26], and condensed tannins from *Delonix regia* [27]. Thus, compounds **5** & **18** were confirmed as condensed tannins.

Compounds **1**, **11**, and **12** were newly isolated and identified as new compounds from the same medicinal plant in the previous report by Nguyen et al. [18]. However, the α-glucosidase inhibitory activity of these compounds was investigated for the first time in this study. They were determined as new aGIs based on the current literature review, and all their NMR spectrums, including ^1^H-MNR, ^13^C-NMR, DEPT_135_, COSY, HSQC, and HMBC were presented in the supplementary materials section (Figures S1–S21). Compound **14**, leonuriside, was reported to possess several bioactive properties, including α-amylase inhibition [18], COX-1 inhibition [24], and potent anti-NO [22]. However, the α-glucosidase inhibition of leonuriside had not been reported before; this compound was also determined as a new aGI.

Other known phenolic compounds: **5**, **9**, **10**, **18**–**20**, and **23**, have been reported to exhibit numerous beneficial bioactivities, including antimicrobial, cytotoxic [28], antiviral [29], antioxidant [30,31], anti-inflammatory [32], and enzyme inhibitory activities [18,20,33,34], as well as exercising a relaxation effect [35]. The results indicate that *Euonymus laxiflorus* Champ. is a rich potent source of hypoglycemic compounds, and possesses many other valuable biological activities.

### 2.3. Comparision of α-Glucosidase Inhibitory Activity of Identified Compounds

To screen the most active α-glucosidase inhibitors, all the isolated inhibitors were tested for their activity at various concentrations; the activity was then expressed as IC_50_ (μg/mL) value. As shown in Table 3, compounds **1**, **5**, **14**, **18**, and **19** demonstrated the most effective inhibition due to their smallest IC_50_ values (0.076–0.926 μg/mL). These inhibitors also showed great maximum inhibition (99–100% at 250 μg/mL). Compounds **10**, **11**, and **12** were also found as potent inhibitors, which possessed low IC_50_ values (11.9–31.6 μg/mL) and high maximum inhibition (87–96%). Compounds **9** and **23** showed weak activity (≤38%). Overall, these novel inhibitors had their activity ranked sequentially based on Duncan’s Multiple Range Test (alpha = 0.01): **9** & **23** ≤ acarbose ≤ **20** ≤ **10**, **11** & **12** ≤ **1**, **5**, **14**, **18** & **19**.

### 2.4. The Effect of Condensed Tannin (CT) on Reducing Plasma Glucose in a Mouse Model

Condensed tannin-ELCTB-3.1 (CT) was isolated at a large amount (~2000 mg) and showed efficient inhibition against α-glucosidase. Thus, this major inhibitor was conducted to test its effect on reducing plasma glucose in mice. To evaluate the effect of the samples on reducing plasma glucose in animals, sucrose and starch tolerance tests were used [11,36]. In this study, starch (3 g/kg bw) was chosen for the assay, since it is abundant in cereals, the daily food of people in Asian countries. Therefore, in the case that the compound (CT) showed significant effect on the reducing plasma glucose in mice, it may show potent hypoglycemic effect in humans.

**Figure 3:**
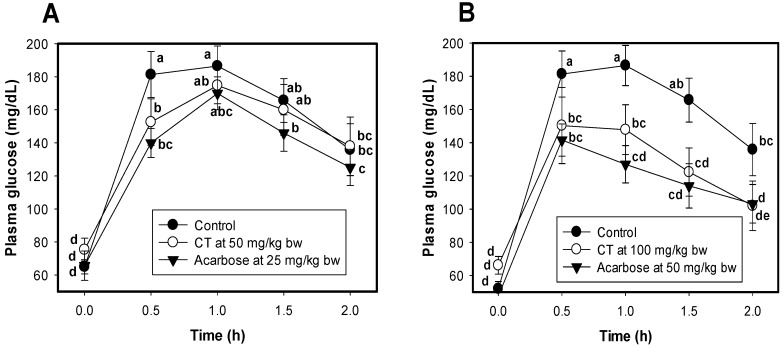
The effect of condensed tannin-ELCTB-3.1.1 and acarbose on reducing plasma glucose in ICR (Institute of Cancer Research) mice. Condensed tannin-ELCTB-3.1.1 and acarbose at the doses of 50 mg/kg bw and 25 mg/kg bw (**A**), 100 mg/kg bw and 50 mg/kg bw (**B**), respectively, were administered to mice (*n* = 8). Distilled water was administered to mice in the control groups (●, *n* = 8). Blood of mice was sampled and measured at 0.5, 1, 1.5, and 2 h after loading the compound or acarbose. Means of blood glucose level with the different letters in the same figure are significantly different based on Duncan’s Multiple Range Test (alpha = 0.05). CV: coefficient of variation.

Two doses of isolated CT (50 and 100 mg/kg bw) were orally administered to mice to evaluate the effect on their plasma glucose level. CT at the dose of 50 mg/kg bw showed significant reduction of plasma glucose in mice at 0.5 h after CT administration; thereafter, the hypoglycemic effect showed less significant difference than that of the control group (mice administered water only) (Figure 3A). On the other hand, the effect of CT on the reduction of plasma of mice at the dose of 100 mg/kg bw was clearly observed from 0.5 to 2 h after CT administration (Figure 3B). Notably, the effect of CT at 100 mg/kg bw was comparable to that of acarbose at 50 mg/kg bw (Figure 3B), and much higher than that of acarbose at 25 mg/kg bw (Figure 3A). The mice showing symptoms of diarrhea during the tests were recorded. CT at all treated doses on mice led to no symptoms of diarrhea in mice, while mice with this illness symptom were recorded at 20% and 50% of the mice treated with acarbose doses of 25 and 50 mg/kg bw, respectively.

Natural products such as tea and coffee with high polyphenolic compounds content [37], were reported to show good effect on the reduction of plasma glucose in mice [38], as well as on postprandial plasma glucose in healthy humans [37]. Some herbal extracts rich in condensed tannins [6,17,39] were also tested for their anti-hyperglycemic effects in diabetic rats. *Psidium littorale* Raddi leaf extract at the dose of 150 mg/kg bw reduced fasting plasma glucose levels in streptozotocin-induced diabetic rats [6]. The methanolic extract of *Euonymus laxiflorus* Champ. trunk bark (the crude sample of CT) showed significant reduction of blood glucose in diabetic rats at the dose of 200 mg/kg bw, comparable to that of acarbose at 120 mg/kg bw [17]. The pinhão coat extract, and the *A. mearnsii* tannin also demonstrated significant effectiveness in diminishing the post-prandial glycemic levels in rats at their doses of 250 mg/kg bw after starch administration [39]. In this study, *Euonymus laxiflorus* Champ. condensed tannins demonstrated significant effect on reducing plasma glucose at their low doses of 50, and 100 mg/kg bw.

**Figure 4:**
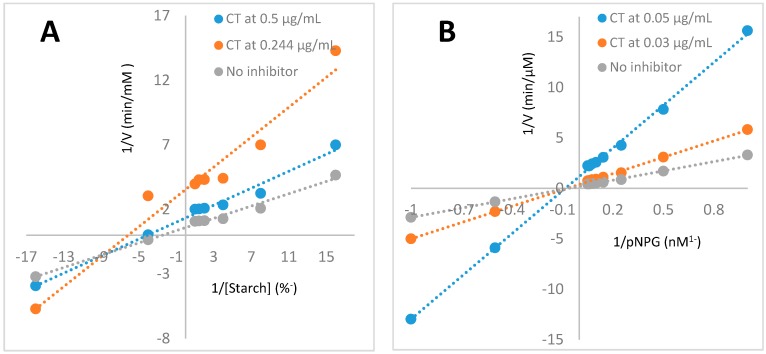
Lineweaver-Burk plots of enzymatic inhibition kinetics of ELC-CT against α-amylase (**A**), and α-glucosidase (**B**).

Condensed Tannins possess vast beneficial bioactivities, including cardio-protective, antioxidative, antitumor, antiviral, antibacterial, immune-modulatory, anti-inflammatory activities, antiobesity, antidiabetic [40,41], and hypoglycemic effects [39]. *Euonymus laxiflorus* Champ. condensed tannins (condensed tannin-ELCTB-3.1) were found to possess potent hypoglycemic effect in this study. It is well known that starch, a polysaccharide, is degraded by amylases to dextrin oligomers; these oligomers are then further degraded to α-d-glucoses by α-glucosidase. These monomeric may enter the blood circulation via intestinal epithelial absorption. Therefore, the use of α-amylase and α-glucosidase inhibitors may block or slow down this process, leading to the hypoglycemic effect after meals. *Euonymus laxiflorus* Champ. condensed tannins (ELC-CT) showed effective inhibition both on α-amylase [16] and α-glucosidase, since this resulted in their significant hypoglycemic effect. To determine the inhibition mode of ELC-CT, three concentrations of ELC-CT: 0.5, 0.244, 0 µg/mL, and 0.05, 0.03, 0 µg/mL against α-amylase, and α-glucosidase, respectively, were tested. According to the Lineweaver-Burk plots of enzymatic inhibition kinetics of ELC-CT against α-amylase (Figure 4A), and α-glucosidase (Figure 4B), ELC-CT was determined as mix (non-competitive-uncompetitive) inhibition against α-amylase, and non-competitive inhibition against α-glucosidase via comparison to the typical Lineweaver-Burk plots [42].

These results indicated that ELC-CT did not combine with enzymes in the active sites of both enzymes (α-amylase, and α-glucosidase); ELC-CT combined with α-glucosidase to produce dead-end enzyme-inhibitor complex regardless of the substrate (pNPG) was bound; while some ELC-CT molecules could combine with α-amylase-starch complex, but did not combine with free amylase, and some ELC-CT molecules could combine with both α-amylase-starch complex, and free amylase to block the amylase activity [42].

According to the results of this study and the previous report [17], suggest that *Euonymus laxiflorus* Champ. is a rich source of condensed tannins, which could be developed as health food with potent hypoglycemic effect.

## 3. Materials and Methods

### 3.1. Materials

The methanolic extract of *Euonymus laxiflorus* Champ. trunk bark was obtained from the previous study [4]. *Saccharomyces cerevisiae* (yeast) α-glucosidase and acarbose were purchased from Sigma Chemical Co., St. Louis City, MO, USA; *p*-nitrophenyl glucopyranoside (*p*NPG) was obtained from Sigma Aldrich, 3050 Spruce Street, St. Louis, MO, USA. All the solvents and common chemicals were obtained at their highest grade.

### 3.2. Biological Activity Assays

#### 3.2.1. α-Glucosidase Inhibitory Activity Determination

The α-glucosidase inhibitory activity was closely detected following the method described in detail by Nguyen et al. (2018) [12]. The mixture of 50 μL α-glucosidase, 50 μL sample solutions, 100 μL buffer was pre-incubated at 37 °C for 20 min; the reaction then started when 50 μL of *p*-nitrophenyl glucopyranoside (10 mmol/L) was added to the mixture. After incubation at the same temperature for 30 min, the reaction was stopped by adding 100 μL Na_2_CO_3_ solution (1 mol/L) to the reaction mixture; the absorbance of this final mixture was then measured at 410 nm (**A**). The control group also underwent the same described method with the use of 50 μL buffer instead of 50 μL sample solutions; the absorbance was recorded at 410 nm (**B**). The aGI activity (%) was calculated using the following equation:

aGI (%) = (A − B)/ A × 100.

The inhibition was also expressed as IC_50_ value determined as per the previous study [2]. The enzyme and the samples were prepared in 0.1 mol/L potassium phosphate buffer (pH 7). The purified compounds were tested at concentrations in the range of 0.015–250 μg/mL and the IC_50_ plots for all tested compounds (**1**, **5**, **10**, **11**, **12**, **14**, **18**, **19**, and **20**), and acarbose were presented in the supplementary materials section (Figures S22–S31). The corresponding % inhibition at each concentration of all tested compounds were also recorded in Figure S32.

#### 3.2.2. Experimental Animal Protocol

Animals: seven week-old male ICR (Institute of Cancer Research) mice were purchased from The National Laboratory Animal Center (No. 128, Sec. 2, Academia Rd., Nangang Dist., Taipei City 11529, Taiwan) and tested in accordance with the approval and guidelines of the Institutional Animal Care and Use Committee of the National Research Institute of Chinese Medicine, Ministry of Health and Welfare (IACUC No. 104-706-1, 29 December 2014). Forty ICR mice were randomly divided into 5 groups (8 mice/group), including a control group orally administered with distilled water, and 4 experimental groups orally administered with 50 mg CT/kg bw, 100 mg CT/kg bw, 25 mg acarbose/kg bw, and 50 mg acarbose/kg bw, respectively. Distilled water was used to prepare the condensed tannins and acarbose solutions.

Assay: The effect of condensed tannin-ELCTB-3.1.1 on the reduction of plasma glucose in mice was performed according to the experimental animal protocol described by Nguyen et al. (2017) [11] with the use of starch solution (3 g/kg bw) instead of sucrose solution administered to ICR mice. All the mice groups were fasted overnight (16 h), and then orally administered water (control group), CT or acarbose (4 experimental groups); thereafter (20 min) starch solution (3 g/kg bw) was orally administered to mice; blood was then sampled and measured after 0.5, 1.0, 1.5, and 2.0 h.

#### 3.2.3. Determination of Enzymatic Inhibition Modes of Isolated Condensed Tannin

The inhibition mode of condensed tannin was determined by performing as the reported assay with minor modification [10]. Enzyme kinetics of the isolated condensed tannin was determined using the α-glucosidase and α-amylase inhibitory activity assay mentioned above. The concentration range used was 2–18 mmol/L pNPG, and 0.0625–2% starch for the α-glucosidase, and α-amylase inhibitory activity assays, respectively. The inhibition modes of the sample were determined by analyzing the Vmax and comparing the Lineweaver-Bur plots of condensed tannin to the standard typical Lineweaver-Bur plots [42].

### 3.3. Purification and Identification Procedures of the Active aGIs

Methanolic extract of **ELCTB** (40 g) was primarily fractionated via a Diaion column with successive eluting with distilled water, 40% MeOH, 70% MeOH, 100% MeOH, and 100% ethyl acetate to obtain 5 fractions: **ELCTB-1** (20.97 g), **ELCTB-2** (9.12 g), **ELCTB-3** (4.67 g), **ELCTB-4** (1.46 g), **ELCTB-5** (1.63 g), respectively.

**ELCTB-2** (4.5 g) was loaded onto an ODS column and 10 subfractions were obtained with the elution of a gradient mobile phase of MeOH in H_2_O (0–100%, *v*/*v*). The 3 subfractions: **ELCTB-2.1** (1.8 g), **ELCTB-2.2** (0.5 g), and **ELCTB-2.3** (0.25 g) were obtained by eluting with 0%, 5%, and 10% MeOH in H_2_O, respectively. All of these subfractions were further separated by injecting them into a preparative HPLC (Pr-HPLC) (preparative Cosmosil 5C18-AR-II column equipped with a 250 × 20 mm i.d. and a UV detector (Nacalai Tesque, Inc., Kyoto, Japan) at 210 and 254 nm) for the isolation of 17 compounds (**1**–**17**) via elution with 8% ACN (compounds **1**–**5**), 5% ACN (compounds **5**–**17**).

**ELCTB-3** (4.5 g) was also loaded onto the same ODS column and then eluted by ACN in the H_2_O mobile phase with the gradient of 10–100%, *v*/*v*, to obtain 10 subfractions (**ELCTB-3.1-ELCTB-3.10**). Two subfractions: **ELCTB-3.1** (1.8 g), and **ELCTB-3.3** (0.6 g) were collected by eluting with 10% and 20% ACN, respectively. **ELCTB-3.3** (0.6 g) was further separated via the same column and eluted with ACN in H_2_O mobile phase with a gradient of 0–100%, *v*/*v*, and 10 subfractions (**ELCTB-3.3.1-ELCTB-3.3.10**) were obtained. Of these, 3 subfractions: **ELCTB-3.3.4**, **ELCTB-3.3.5**, and **ELCTB-3.3.6**, eluted with 10%, 13%, and 16% ACN, respectively, were injected into the Pr-HPLC for the isolation of 8 compounds (**19**–**26**) by the elution with 14% ACN (**19**, **20**, **25**, **26**), and 22% MeOH (**21**–**24**). Compound **18** was obtained from subfraction **ELCTB-3.1** via dialysis. The purification process is summarized in Figure 1.

The chemical structures of the isolated compounds were identified via analysis of their NMR data, coupled with the comparison of those of reported compounds. The ^1^H and ^13^C-NMR spectra, and 2D-NMR spectra (COSY, HMQC, HMBC, and NOESY), were recorded in MeOH-*d*_4_ on a Bruker AVX NMR spectrometer (Bruker, Karlsruhe, Germany) operating at 600 MHz for 1 to 12 h and 150 MHz for ^13^C using the MeOH-*d*_4_ solvent peak as internal standard (δ_H_ 3.317, δ_C_ 49.1 ppm).

### 3.4. Characteristics and NMR Data of Identified Compounds

*Compound **1**: Walterolactone A*/*B β-d-pyranoglucoside* was obtained as a white amorphous powder. ^1^H-NMR data (600 MHz, MeOH-*d*_4_, δ_H_ ppm): 5.86 (q, *J* = 1.8 Hz), 4.57 (dd, *J* = 12.0, 3.0 Hz), 4.42 (dd, *J* = 12.0, 3.0 Hz), 4.41 (d, *J* = 7.8 Hz), 4.40 (t, *J* = 3.0 Hz), 3.88 (dd, *J* = 12.0, 1.8 Hz), 3.67 (dd, *J* = 12.0, 5.4 Hz), 3.36 (t, *J* = 9.0 Hz), 3.29 (m, 2 H), 3.17 (dd, *J* = 9.0, 7.8 Hz), 2.10 (d, *J* = 1.8 Hz). ^13^C-NMR data (150 MHz, MeOH-*d*_4_, δ_C_ ppm): 166.1, 158.6, 118.9, 103.0, 78.2, 78.0, 74.8, 71.5, 71.0, 70.3, 62.7, 20.4.

*Compound **9**: 1-β-d-glucopyranosyloxy-3,5-dimethoxy-4-hydroxybenzene* was obtained as a white amorphous powder. ^1^H-NMR data (600 MHz, MeOH-*d*_4_, δ_H_ ppm): 6.5 (s, 2H), 4.75 (d, *J* = 7.2 Hz), 3.91 (dd, *J* = 12.0, 2.4 Hz), 3.81 (s, 6 H), 3.66 (dd, *J* = 12.0, 6.6 Hz), 3.4 ~3.5 (m, 3 H). ^13^C-NMR data (150 MHz, MeOH-*d*_4_, δ_C_ ppm): 152.4, 149.4, 131.0, 103.8, 96.6, 78.2, 77.9, 74.9, 62.7and 56.8.

*Compound **10**: (−)-Gallocatechin* was obtained as a white amorphous powder. ^1^H-NMR data (600 MHz, MeOH-*d*_4_, δ_H_ ppm): 6.40 (s, 2H), 5.92 (d, *J* = 2.4 Hz), 5.85 (d, *J* = 2.4 Hz), 4.52 (d, *J* = 7.2 Hz), 3.96 (m), 2.8 (dd, *J* = 15.6, 5.4 Hz), 2.49 (dd, *J* = 15.6, 7.8 Hz). ^13^C-NMR data (150 MHz, MeOH-*d*_4_, δ_C_ ppm): 157.8, 157.6, 156.8, 146.9, 134.0, 131.5, 107.2, 100.8, 96.3, 95.5, 82.9, 68.7, 28.1.

*Compound **11**: Schweinfurthinol 9-O-β-d-pyranoglucoside or 1-(4-hydroxyphenyl)-2,3-dihydroxypropan-1-one 3-O-β-d-pyranoglucoside* was obtained as a white amorphous powder. ^1^H-NMR data (600 MHz, MeOH-*d*_4_, δ_H_ ppm): 7.93 (d, *J* = 7.2 Hz, 2H), 6.85 (d, *J* = 7.2 Hz, 2H), 5.28 (dd, *J* = 6.6, 3.6 Hz), 4.25 (d, *J* = 7.8 Hz), 4.18 (dd, *J* = 11.4, 3.6 Hz), 3.84 (dd, *J* = 12.0, 1.8 Hz), 3.73 (dd, *J* = 11.4, 6.6 Hz), 3.65 (dd, *J* = 12.0, 6.0 Hz), 3.32 (overlapped), 3.28 (t, *J* = 9.0 Hz), 3.26 (m), 3.19 (dd, *J* = 9.0, 7.2 Hz). ^13^C-NMR data (150 MHz, MeOH-*d*_4_, δ_C_ ppm): 198.6, 162.8, 132.5, 127.5, 116.6, 105.0, 78.0, 77.8, 75.1, 74.3, 73.8, 71.5, 62.6.

*Compound **12**: 1-O-(3-methyl)-butenoyl-myo-inositol or Myo-inositol 1-O-3,3-dimethylacrylate* was obtained as a white amorphous powder. ^1^H-NMR data (600 MHz, MeOH-*d*_4_, δ_H_ ppm): 5.81 (brs), 4.62 (dd, *J* = 10.2, 2.4 Hz), 4.05 (t, *J* = 2.4 Hz), 3.82 (dd, *J* = 10.2, 9.6 Hz), 3.63 (t, *J* = 9.0 Hz), 3.41 (dd, *J* = 9.0, 2.4 Hz), 3.22 (dd, *J* = 9.6, 9.0 Hz), 2.16 (d, *J* = 1.2 Hz), 1.92 (d, *J* = 1.2 Hz). ^13^C-NMR data (150 MHz, MeOH-*d*_4_, δ_C_ ppm): 167.7, 158.7, 117.0, 76.6, 75.0, 74.1, 73.1, 72.0, 71.8, 27.4, 20.4.

*Compound **14**: Leonuriside* was obtained as a white amorphous powder. ^1^H-NMR data (600 MHz, MeOH-*d*_4_, δ_H_ ppm): 6.13 (s, 2H), 4.66 (d, *J* = 7.2 Hz), 3.79 (s, 6 H), 3.78 (dd, *J* = 12.0, 2.4 Hz), 3.67 (dd, *J* = 12.0, 5.4 Hz), 3.4~3.5 (m, 2 H), 3.36 (overlapped), 3.19 (m). 13C-NMR data (150 MHz, MeOH-d4, δC ppm): 155.9, 154.7, 129.6, 94.5, 106.1, 78.2, 77.7, 75.7, 65.2 and 56.8.

*Compounds **5** & **18**: condensed tannins* were obtained as brow yellow amorphous powder: ^13^C-NMR data (150 MHz, MeOH-*d*_4_, δ_C_ ppm): 161.0, 155.0, 144.5, 138.5, 132.0, 122.0–118.0, 116.3–114.0, 107.0, 102.0, 97.0, 76.0, 35.5 and 34.0).

*Compound **19** (+)-Catechin* was obtained as a white amorphous powder. ^1^H-NMR data (600 MHz, MeOH-*d*_4_, δ_H_ ppm): 6.83 (d, *J* = 1.8 Hz), 6.76 (d, *J* = 7.8 Hz), 6.71 (dd, *J* = 7.8, 1.8 Hz), 5.93 (d, *J* = 2.4 Hz), 5.85 (d, *J* = 2.4 Hz), 4.46 (d, *J* = 7.8 Hz), 3.98 (m), 2.83 (dd, *J* = 16.2, 5.4 Hz), 2.49 (dd, *J* = 16.2, 8.4 Hz). ^13^C-NMR data (150 MHz, MeOH-*d*_4_, δ_C_ ppm): 157.7, 157.5, 156.8, 146.2, 132.2, 120.1, 116.2, 115.3, 100.9, 96.3, 95.5, 82.8, 68.7, 28.5.

*Compound **20**: Methyl galloate* was obtained as a white amorphous powder. ^1^H-NMR data (600 MHz, MeOH-*d*_4_, δ_H_ ppm): 7.05 (s, 2H), 3.72 (s, 3H). ^13^C-NMR data (150 MHz, MeOH-*d*_4_, δ_C_ ppm): 167.7, 145.9, 138.8, 121.0, 109.5, 52.0.

*Compound **23**: (−)-Catechin* was obtained as a white amorphous powder. ^1^H-NMR data (600 MHz, MeOH-*d*_4_, δ_H_ ppm): 6.83 (d, *J* = 1.8 Hz), 6.76 (d, *J* = 7.8 Hz), 6.71 (dd, *J* = 7.8, 1.8 Hz), 5.93 (d, *J* = 2.4 Hz), 5.85 (d, *J* = 2.4 Hz), 4.46 (d, *J* = 7.8 Hz), 3.98 (m), 2.83 (dd, *J* = 16.2, 5.4 Hz), 2.49 (dd, *J* = 16.2, 8.4 Hz). ^13^C-NMR data (150 MHz, MeOH-*d*_4_, δ_C_ ppm): 157.7, 157.5, 156.8, 146.2, 132.2, 120.1, 116.2, 115.3, 100.9, 96.3, 95.5, 82.8, 68.7, 28.5.

### 3.5. Statistical Analysis

Statistical Analysis Software (SAS) version 9.4, provided by SAS Institute Taiwan Ltd., Minsheng East Road, Section 2, Taipei, Taiwan 149-8, was used to analyze the differences between the means of inhibition, and blood glucose level via Duncan’s Multiple Range Test (alpha = 0.01 or 0.05). All tests were repeated in triplicate.

## 4. Conclusions

Eleven hypoglycemic compounds were isolated and identified from the methanolic extract of *Euonymus laxiflorus* Champ. trunk bark. Of these, four novel compounds of walterolactone A/B β-d-pyranoglucoside (**1**), schweinfurthinol 9-*O*-β-d-pyranoglucoside (**11**), 1-*O*-(3-methyl)-butenoyl-myo-inositol (**12**), and leonuriside (**14**) were determined as new α-glucosidase inhibitors. The results of in vitro tests indicated that most of the isolated compounds (**1**, **5**, **10**–**12**, **17**, and **18**) showed much higher activity than that of acarbose. Codensed tannin (**18**) demonstrated significant reduction of blood glucose in mice at the dose of 100 mg/kg bw. The results could enrich the current biological activities of constituents isolated from *Euonymus laxiflorus* Champ. species, and also suggest that this medicinal plant is a valuable source of bioactive compounds for development as health food or drugs with potent hypoglycemic effect.

## Figures and Tables

**Table 1:** α-Glucosidase inhibition by some MeOH extracts of medicinal plants collected in Dak Lak.

Scientific Name of Medicinal Plants	Part Used	IC_50_ (µg/mL)	Ref.
*Euonymus laxiflorus* Champ.	Trunk bark	360 ± 28.9 ^d,e^	Nguyen et al., 2017 [4]
*Euonymus laxiflorus* Champ.	Leaves	670 ± 40.4 ^d^	Nguyen et al., 2017 [4]
*Cinnamomum cassia J. S. Presl.*	Trunk bark	1080 ± 103.9 ^c^	Nguyen et al., 2017 [4]
*Terminalia bellirica*	Leaves	660 ± 63.5 ^d^	Nguyen et al., 2017 [4]
*Terminalia bellirica*	Trunk bark	410 ± 30.4 ^d,e^	Nguyen et al., 2016 [5]
*Terminalia corticosa*	Trunk bark	1420 ± 20.2 ^b,c^	Nguyen et al., 2016 [5]
*Psidium littorale Raddi*	Leaves	250 ± 10.4 ^e^	Nguyen et al., 2018 [6]
*Dalbergia tonkinensis*	Heartwood	1720 ± 116 ^b^	Nguyen et al., 2018 [7]
*Dalbergia tonkinensis*	Trunk bark	2910 ± 289 ^a^	Nguyen et al., 2018 [7]
*Dalbergia tonkinensis*	Leaves	2780 ± 173 ^a^	Nguyen et al., 2018 [7]

α-Glucosidase from rat was used for testing; results are means ± SD of multi tests (*n* = 3); coefficient of variation = 12.35314; the means of IC_50_ values with the different letter are significantly different in comparison based on Duncan′s multiple range test (alpha = 0.01) using SAS version 9.4, Statistical Analysis Software analysis.

**Table 2:** α-Glucosidase inhibitory activity of methanol extract of *Euonymus laxiflorus* Champ. trunk bark (ELCTB), and its fraction and subfraction separation via Diaion and ODS (Octadecylsilane) columns, respectively.

Fractions/Subfractions	% Gradient of Solvent in Water	α-Glucosidase Inhibitory Activity	
		IC_50_ (μg/mL)	Inhibition (%) *
Separation of **ELCTB** via a Diaion column with MeOH in H_2_O			
ELCTB		7.16 ± 0.21	98 ± 2.5
ELCTB-1	0	21.75 ± 1.32	67 ± 3.3
ELCTB-2	40	2.80 ± 0.03	99 ± 3.1
ELCTB-3	70	3.50 ± 0.12	98 ± 1.4
ELCTB-4	100	18.50 ± 1.39	97 ± 2.3
ELCTB-5	100% EA	-	-
Separation of **ELCTB-2** via an ODS column with MeOH in H_2_O			
ELCTB-2.1	0	6.20 ± 0.43	97 ± 3.5
ELCTB-2.2	5	85.30 ± 4.03	97 ± 2.9
ELCTB-2.3	10	26.03 ± 1.83	98 ± 1.8
ELCTB-2.4	15	14.96 ± 0.78	98 ± 3.0
ELCTB-2.5	20	6.56 ± 0.45	97 ± 2.0
ELCTB-2.6	25	6.92 ± 0.73	99 ± 1.3
ELCTB-2.7	30	72.95 ± 3.93	98 ± 1.0
ELCTB-2.8	35	6.42 ± 0.41	98 ± 2.8
ELCTB-2.9	40	10.75 ± 0.63	95 ± 3.7
ELCTB-2.10	100	-	-
Separation of **ELCTB-3** via an ODS column with ACN in H_2_O			
ELCTB-3.1	10	1.12 ± 0.03	100 ± 2.3
ELCTB-3.2	15	6.21 ± 0.05	97 ± 2.0
ELCTB-3.3	20	2.48 ± 0.07	97 ± 2.4
ELCTB-3.4	25	1.64 ± 0.04	99 ± 3.6
ELCTB-3.5	30	1.95 ± 0.08	97 ± 2.7
ELCTB-3.6	35	1.89 ± 0.0	100 ± 2.0
ELCTB-3.7	40	4.87 ± 0.12	99 ± 3.0
ELCTB-3.8	45	4.34 ± 0.21	99 ± 3.4
ELCTB-3.9	50	7.506 ± 0.32	96 ± 3.8
ELCTB-3.10	100	-	-
*Acarbose*		1239 ± 78	64 ± 2.4

(-): no α-glucosidase inhibition; (*): the inhibition of samples and acarbose were tested at their concentration of 150 μg/mL and 2500 μg/mL, respectively; EA: ethyl acetate.

**Table 3:** α-Glucosidase inhibitory activity of isolated compounds.

No.	Compound	IC_50_ (μg/mL)	Maximum Inhibition (%)
1	Walterolactone A/B β-d-pyranoglucoside	0.907 ± 0.102 ^e^	100 ± 5.8 ^a^
5	Condensed tannin-ELCTB-2.1.2	0.083 ± 0.004 ^e^	100 ± 6.9 ^a^
9	1-β-d-Glucopyranosyloxy-3,5-dimethoxy-4-hydroxybenzene	UD	35 ± 2.9 ^c^
10	(−)-Gallocatechin	11.9 ± 1.674 ^d^	93 ± 2.9 ^a^
11	Schweinfurthinol 9-*O*-β-d-pyranoglucoside	31.6 ± 0.924 ^b^	87 ± 4.0 ^a^
12	1-*O*-(3-Methyl)-butenoyl-myo-inositol	27.1 ± 1.212 ^c^	96 ± 6.9 ^a^
14	Leonuriside	0.926 ± 0.043 ^e^	99 ± 6.4 ^a^
18	Condensed tannin-ELCTB-3.1.	0.076 ± 0.008 ^e^	100 ± 6.2 ^a^
19	(+)-Catechin	0.113 ± 0.008 ^e^	100 ± 5.9 ^a^
20	Methyl galloate	110 ± 1.732 ^a^	58 ± 1.2 ^b^
23	(−)-Catechin	UD	38 ± 4.0 ^c^
	Acarbose (positive control)	1345 ± 89	65 ± 2.7
	Coefficient of variation (%)	6.510292	8.746013

All compounds were tested at concentrations in the range of 0.122–7.81 μg/mL (compounds **1** and **14**), 0.0152–0.977 μg/mL (compounds **5**, **18**, and **19**), 31.25–250 μg/mL (compounds **9**, **20**, and **23**), 1.95–250 μg/mL (compounds **10**, **11** and **12**), 156.25–2500 μg/mL (acarbose), and the maximum inhibition was recorded at the compounds concentration of 7.81 μg/mL (compounds **1** and **14**), 0.977 μg/mL (compounds **5**, **18**, and **19**), 250 μg/mL (compounds **9**, **10**, **11**, **12**, **20**, and **23**), and 2500 μg/mL (acarbose); the means of IC_50_, and maximum inhibition values with the different letters in the same column are significantly different in comparison based on Duncan’s multiple range test (alpha = 0.01) using SAS version 9.4, Statistical Analysis Software. UD: unable to determine.

## Supplementary Materials

The following are available online, including 32 figures (Figure S1–S32).
