# Supplementary material for: Novel Potent Hypoglycemic Compounds from Euonymus laxiflorus Champ. and Their Effect on Reducing Plasma Glucose in an ICR Mouse Model
Source: Molecules. 2018 Aug 2;23(8):1928. doi: 10.3390/molecules23081928 (PMC6222451; doi:10.3390/molecules23081928)
Supplement: Supplementary file 1 [file molecules-23-01928-s001.pdf]

Supplementary materials:

1. NMR spectrums of 3 new α-glucosidase inhibitors isolated from methanolic extract of Euonymus laxiflorus Champ. trunk bark

1.1. NMR spectrums of Compound 1, Walterolactone A/B β-D-pyranoglucoside: Figure S1 – Figure S7

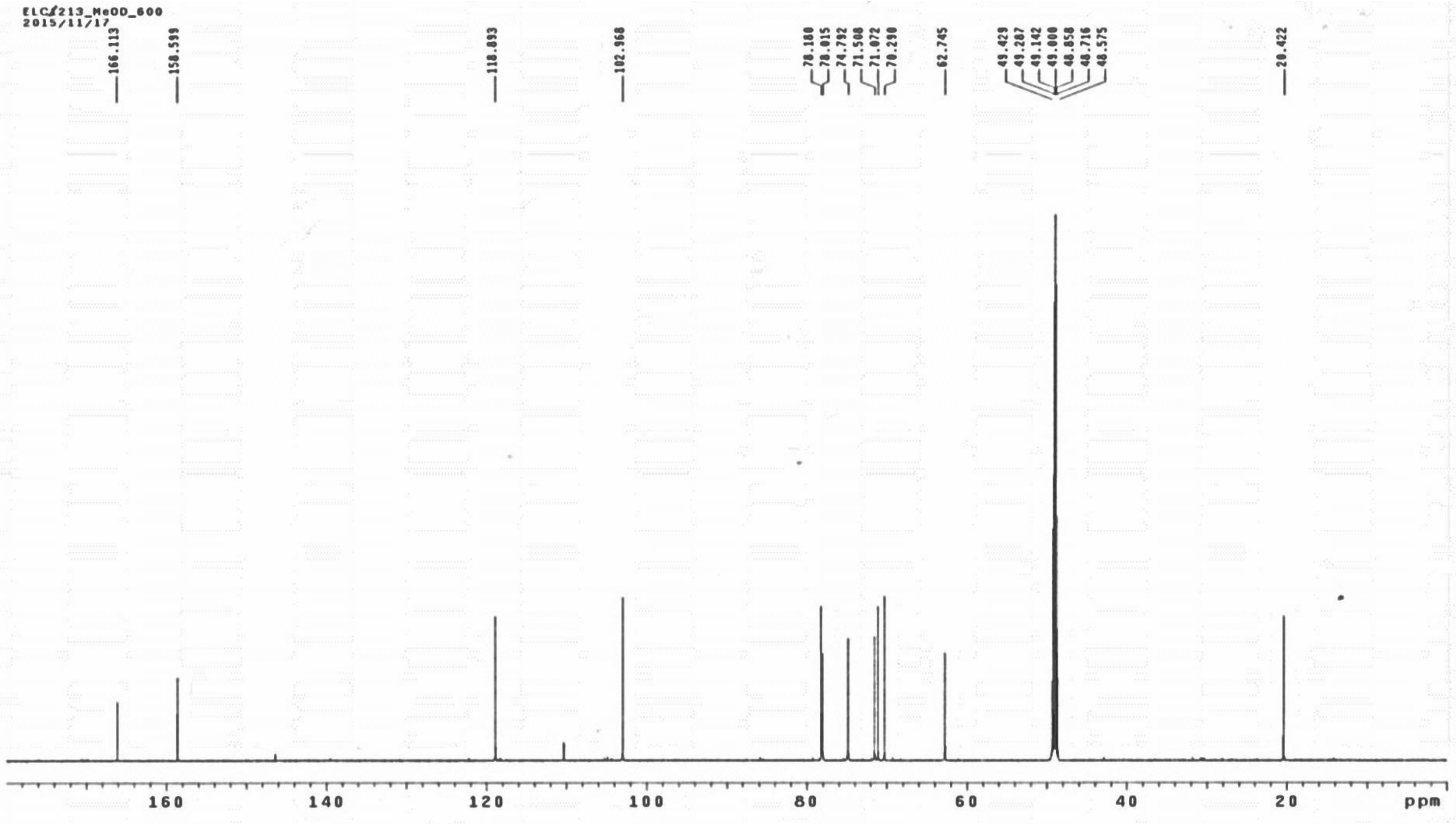

Figure S1. ^{13}C-NMR spectrum of compound **1**, measured in MeOH-d_4 at 150 MHz

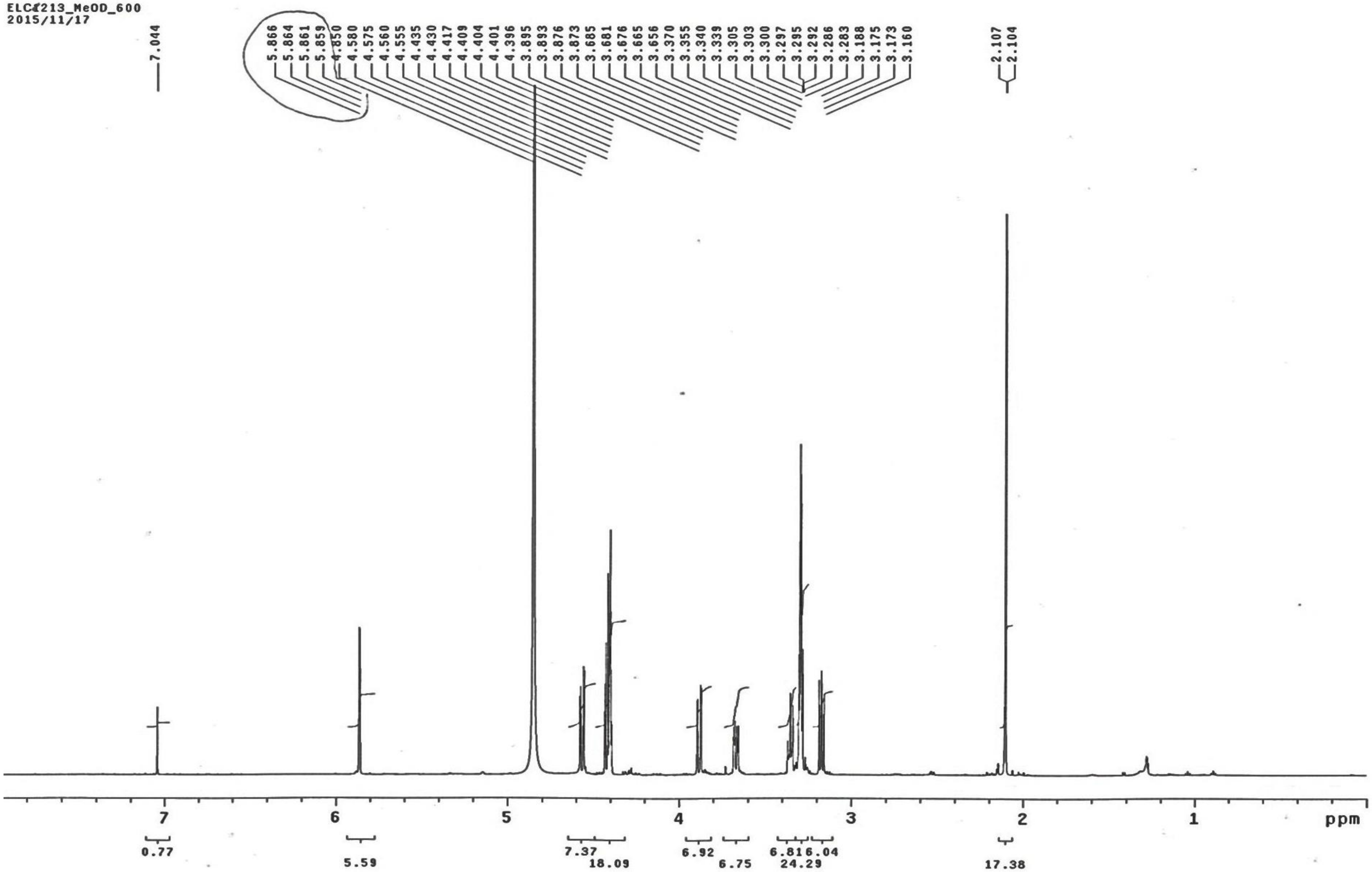

Figure S2. ^{1}H NMR spectrum of compound **1**, measured in MeOH-d_4 at 600 MHz.

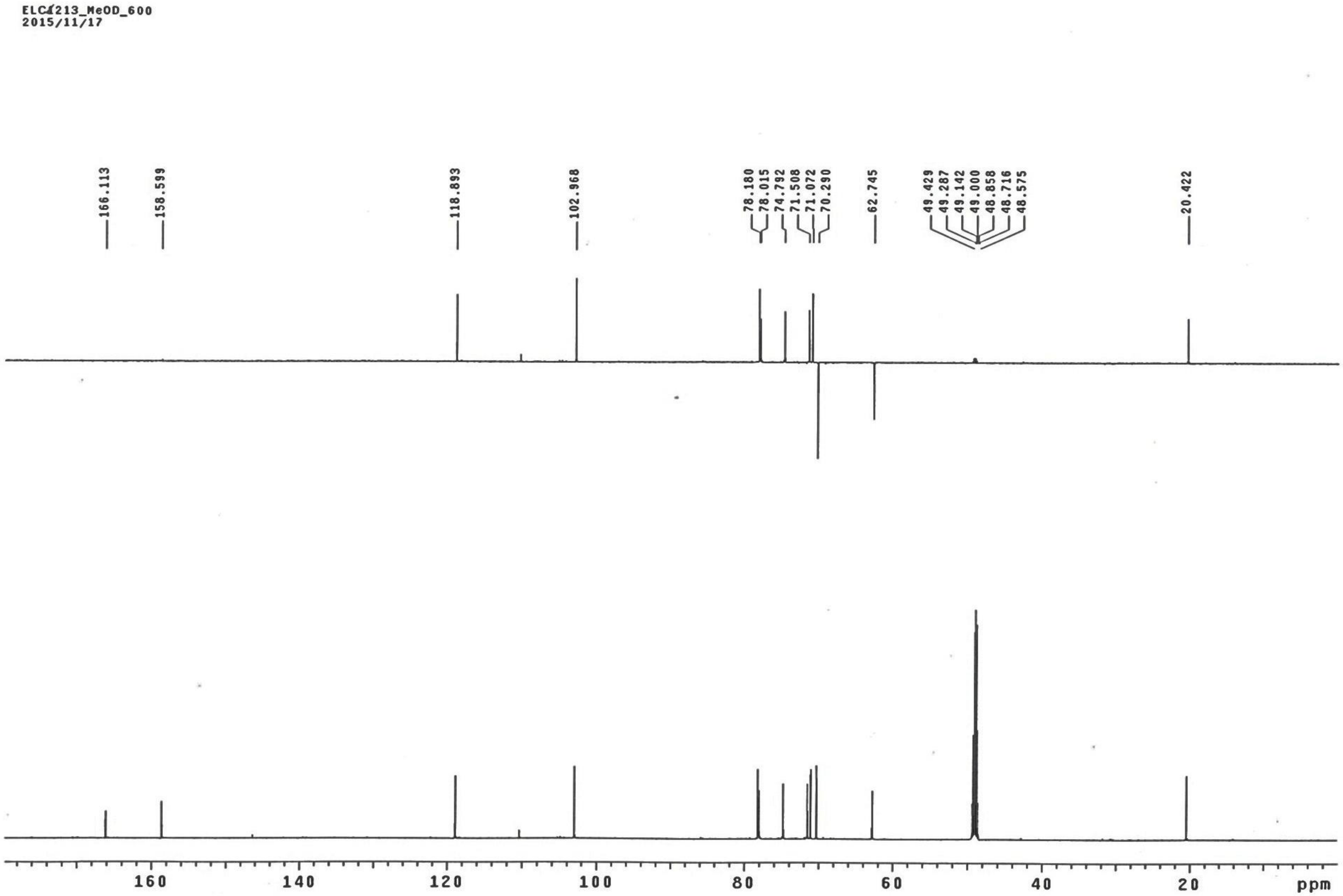

Figure S3. DEPT135 spectrum of compound **1**, measured in MeOH-d_4 at 150 MHz

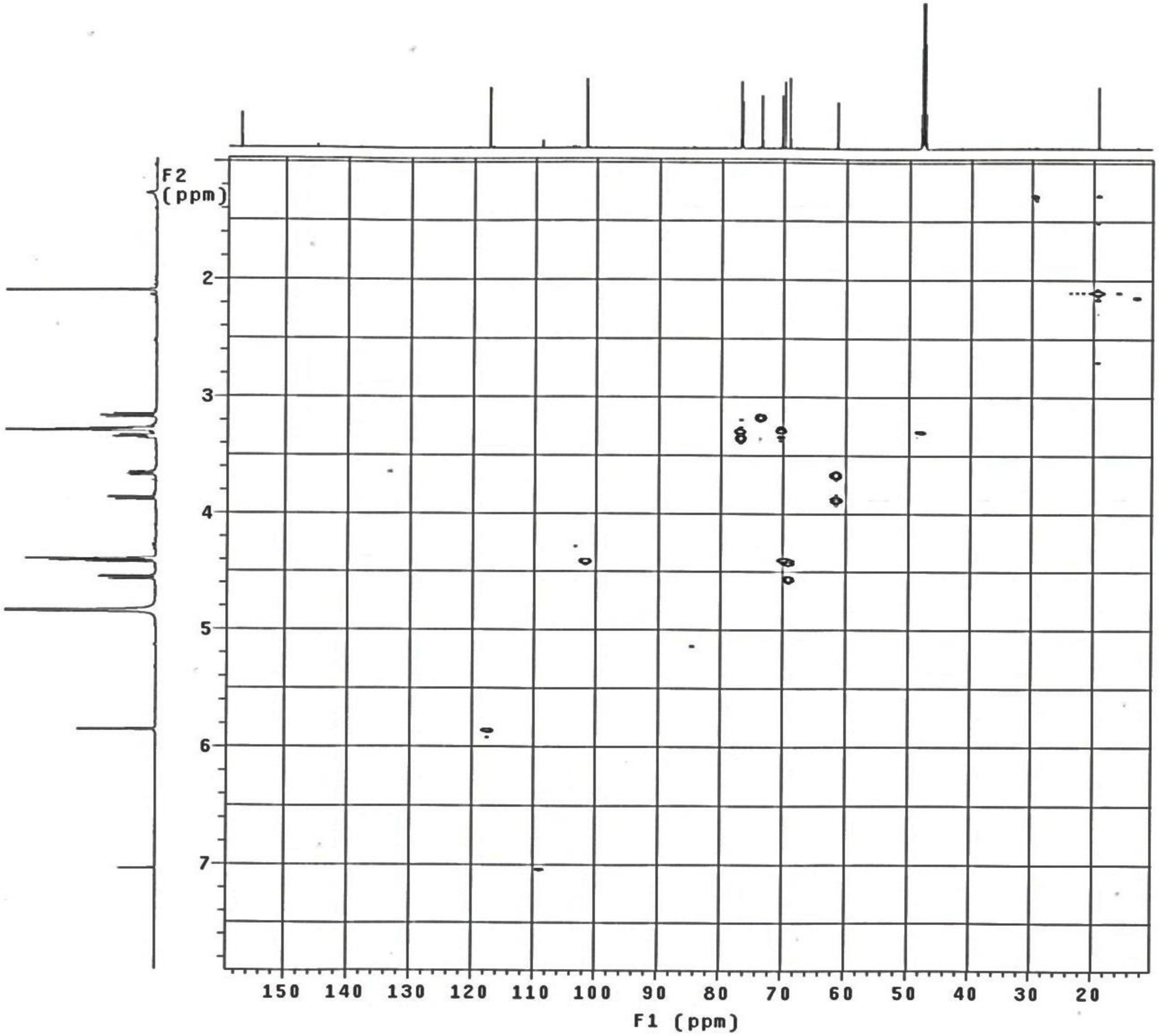

Figure S4. gHSQCAD spectrum of compound **1**, measured in MeOH-d_4

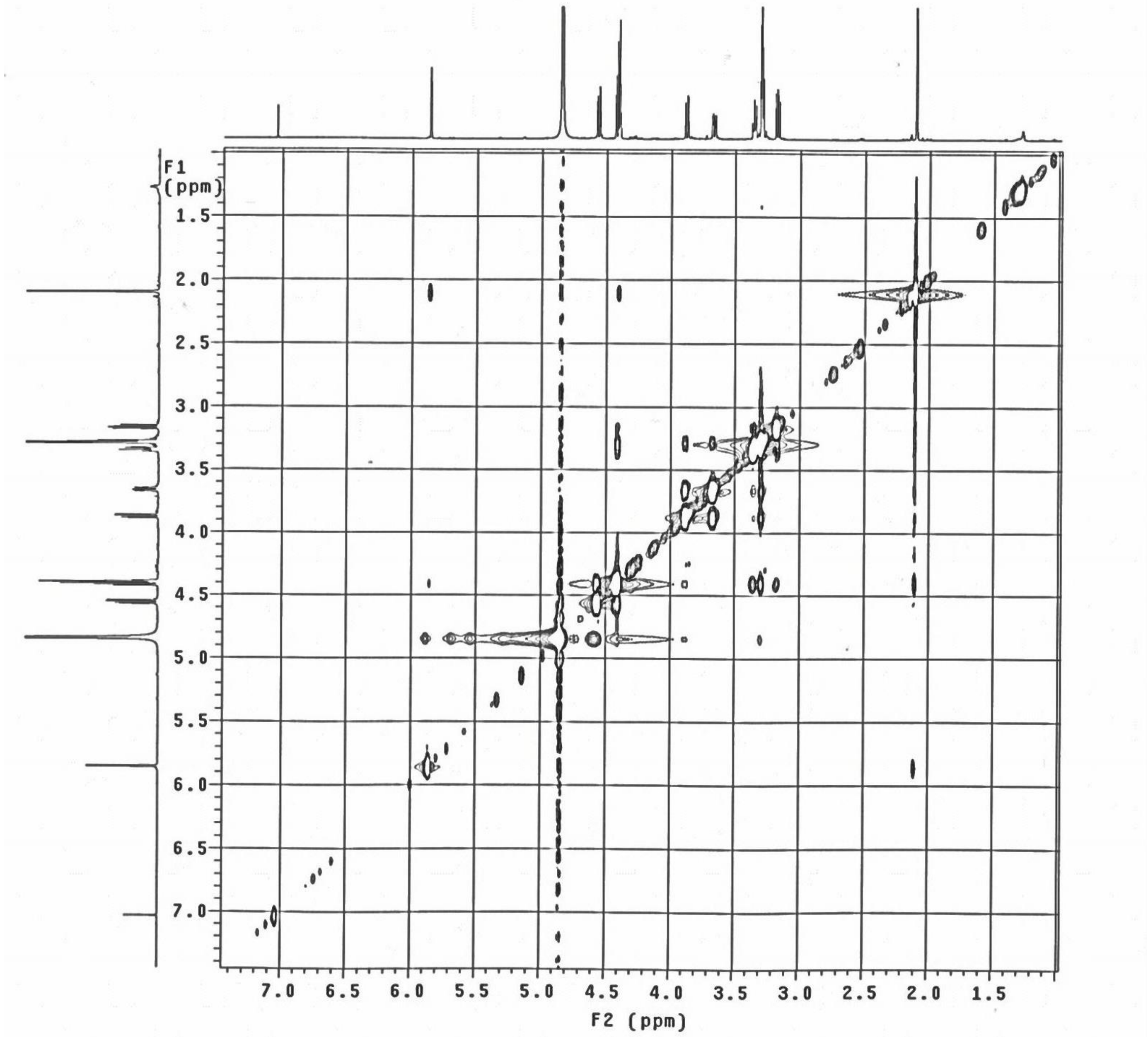

Figure S5. NOESY spectrum of compound **1**, measured in MeOH-d_4

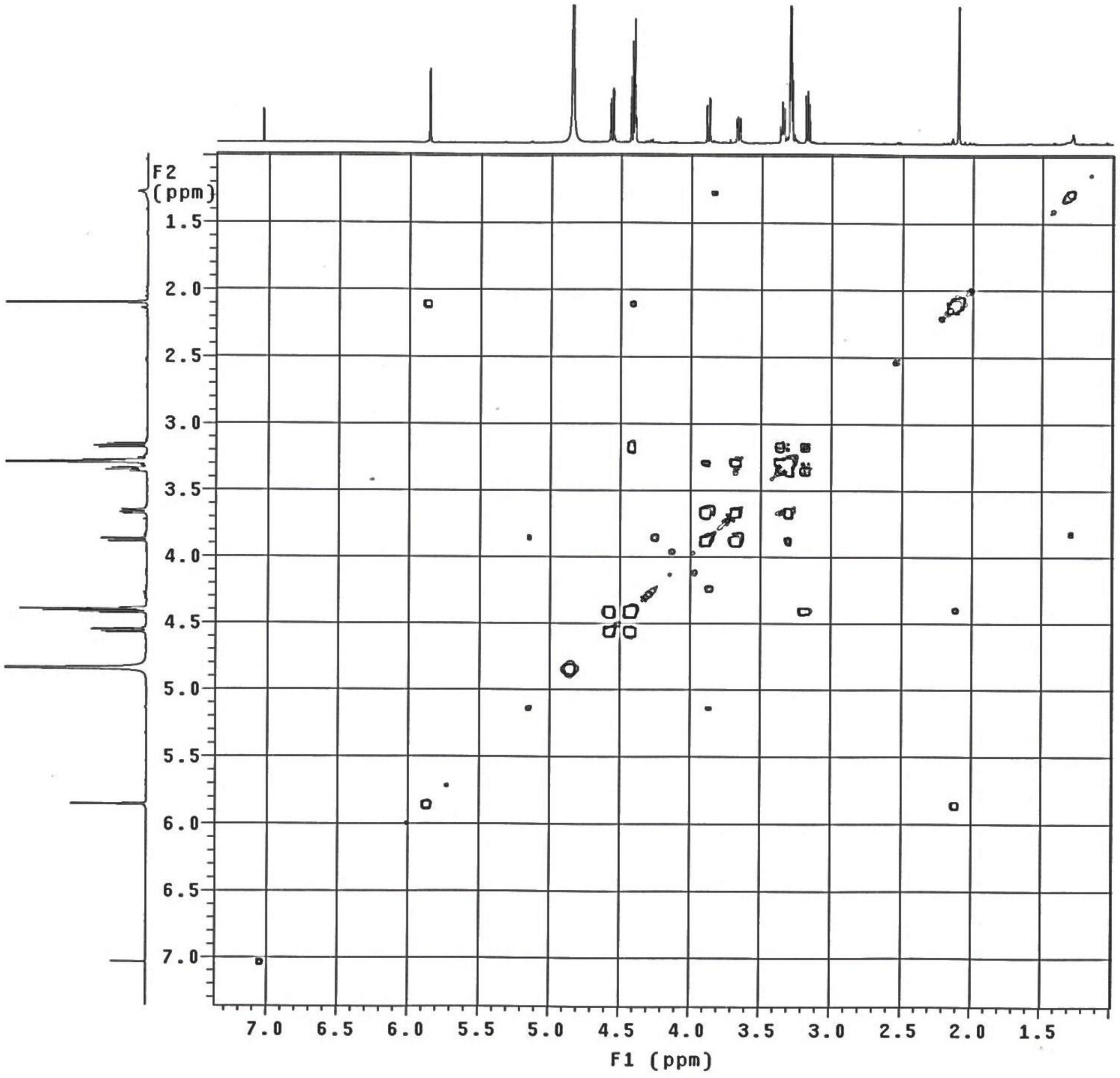

Figure S6. gCOSY spectrum of compound **1,** measured in MeOH-d_4

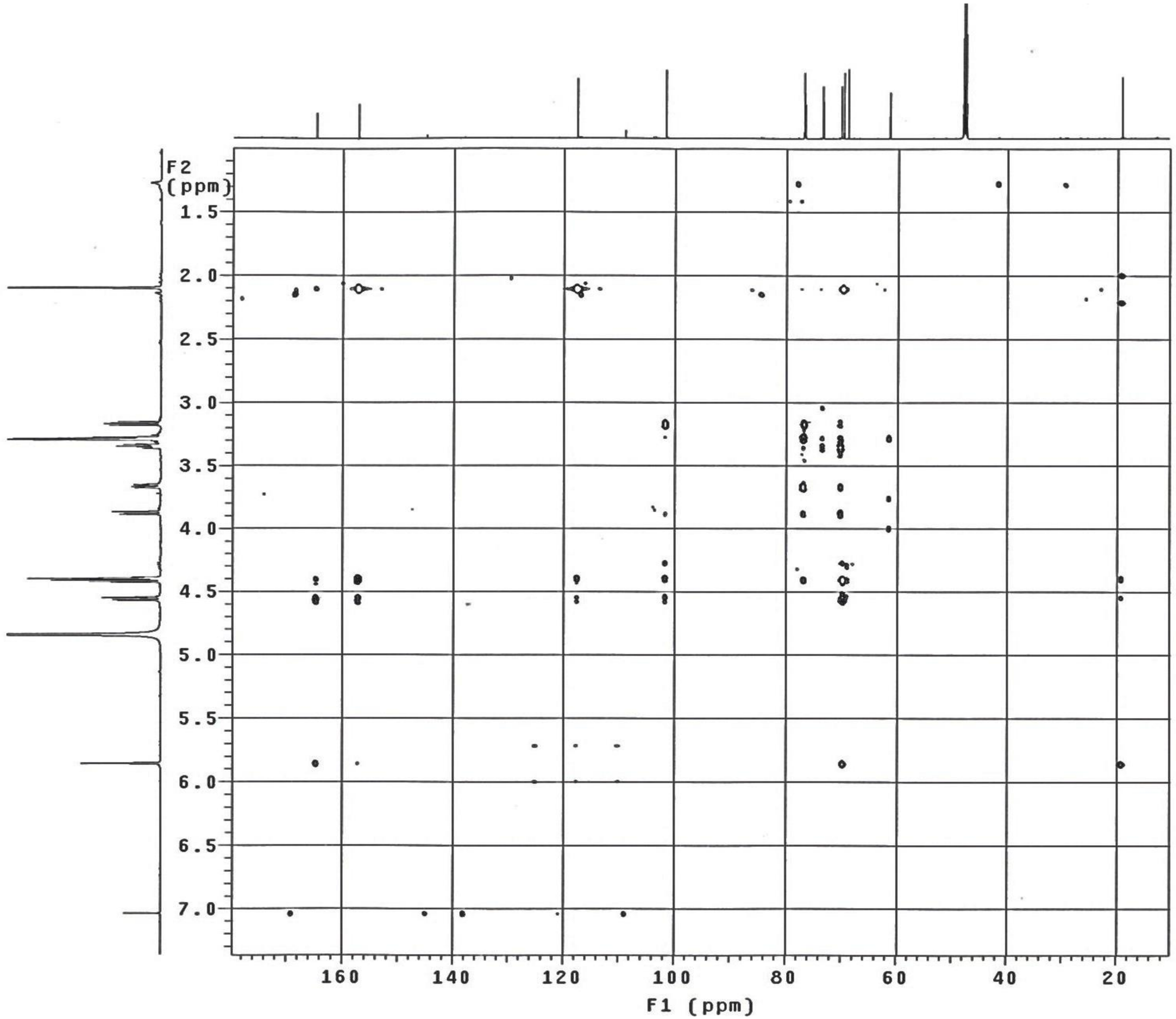

Figure S7. gHMBCAD spectrum of compound **1**, measured in MeOH-d_4

1.2. NMR spectrums of Compound 11, Schweinfurthinol 9-*O*-β-D-pyranoglucoside: Figure S8 – Figure S14

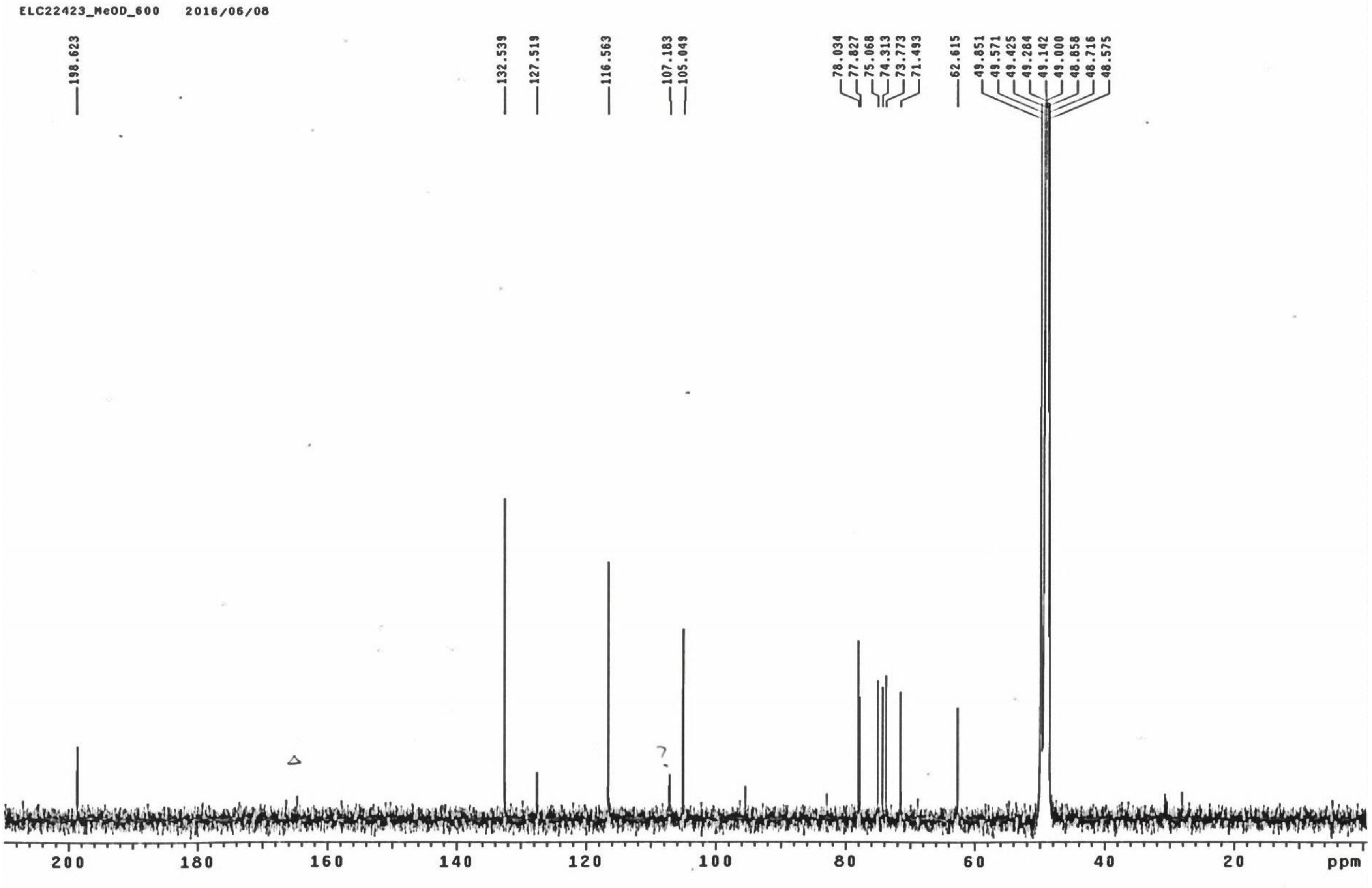

Figure S8. ^{13}C-NMR spectrum of compound **11**, measured in MeOH-d_4 at 150 MHz.

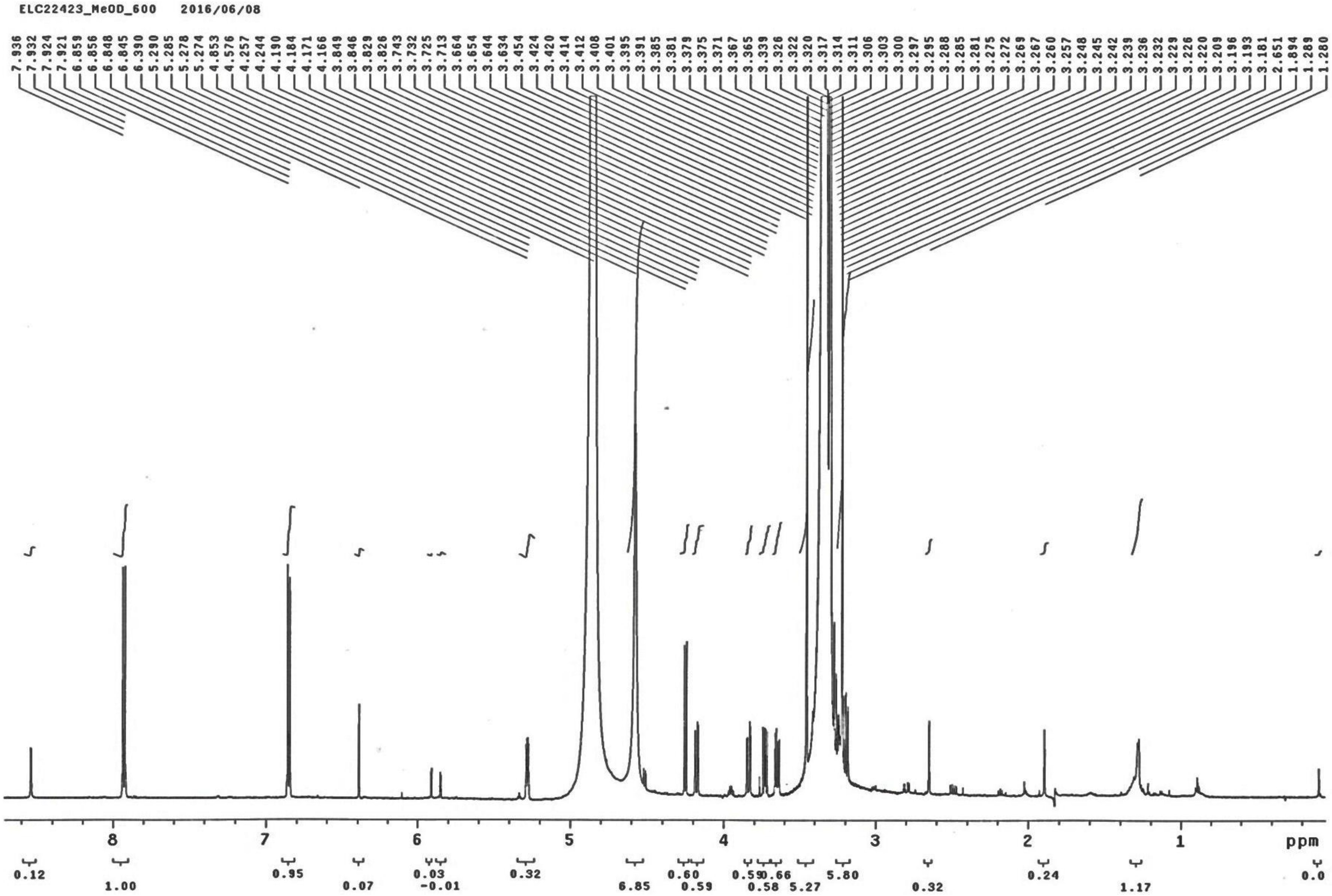

Figure S9. ^{1}H NMR spectrum of compound **11**, measured in MeOH-d_4 at 600 MHz

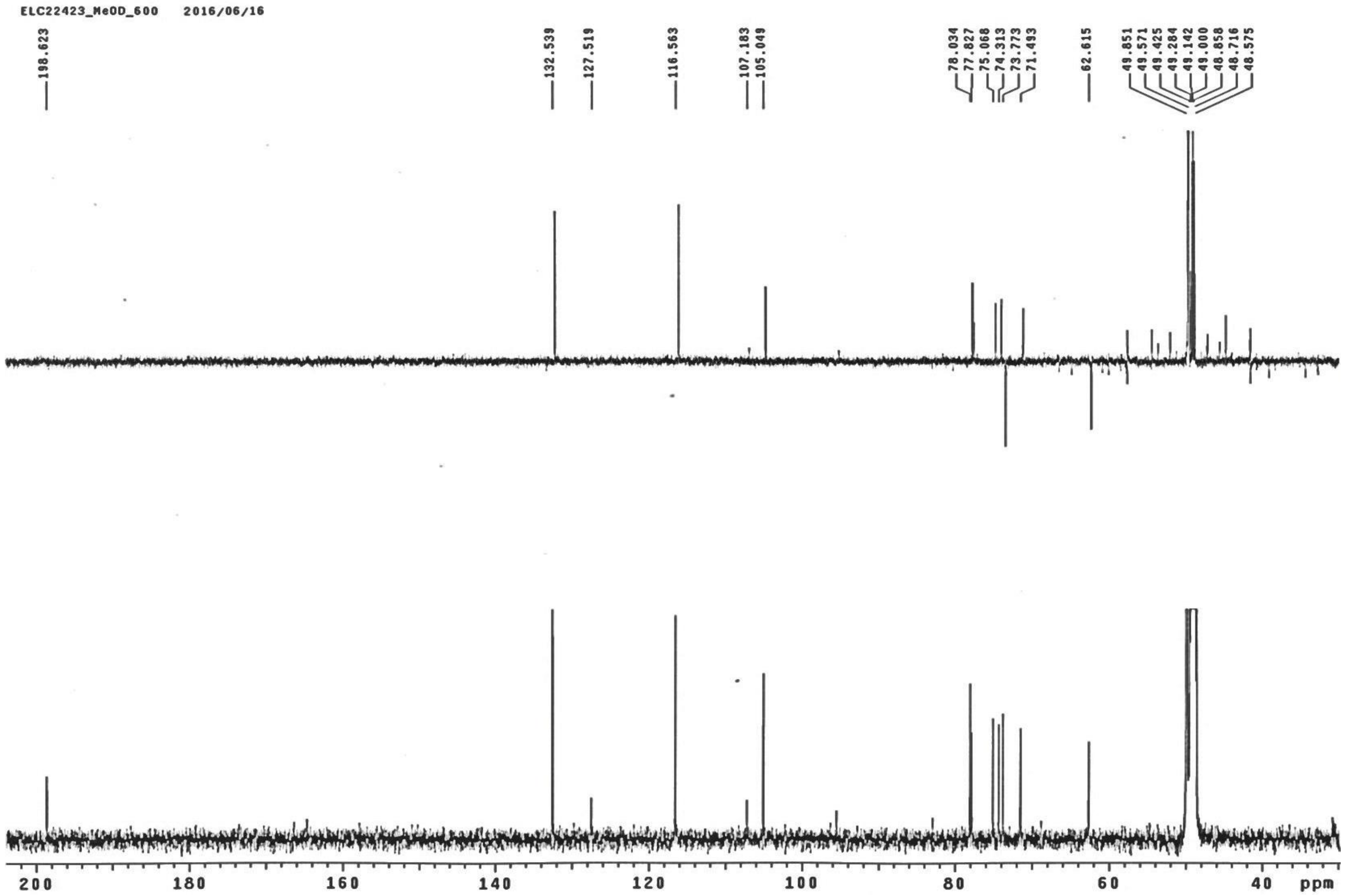

Figure S10. DEPT135 spectrum of compound **11**, measured in MeOH-d_4 at 150 MHz

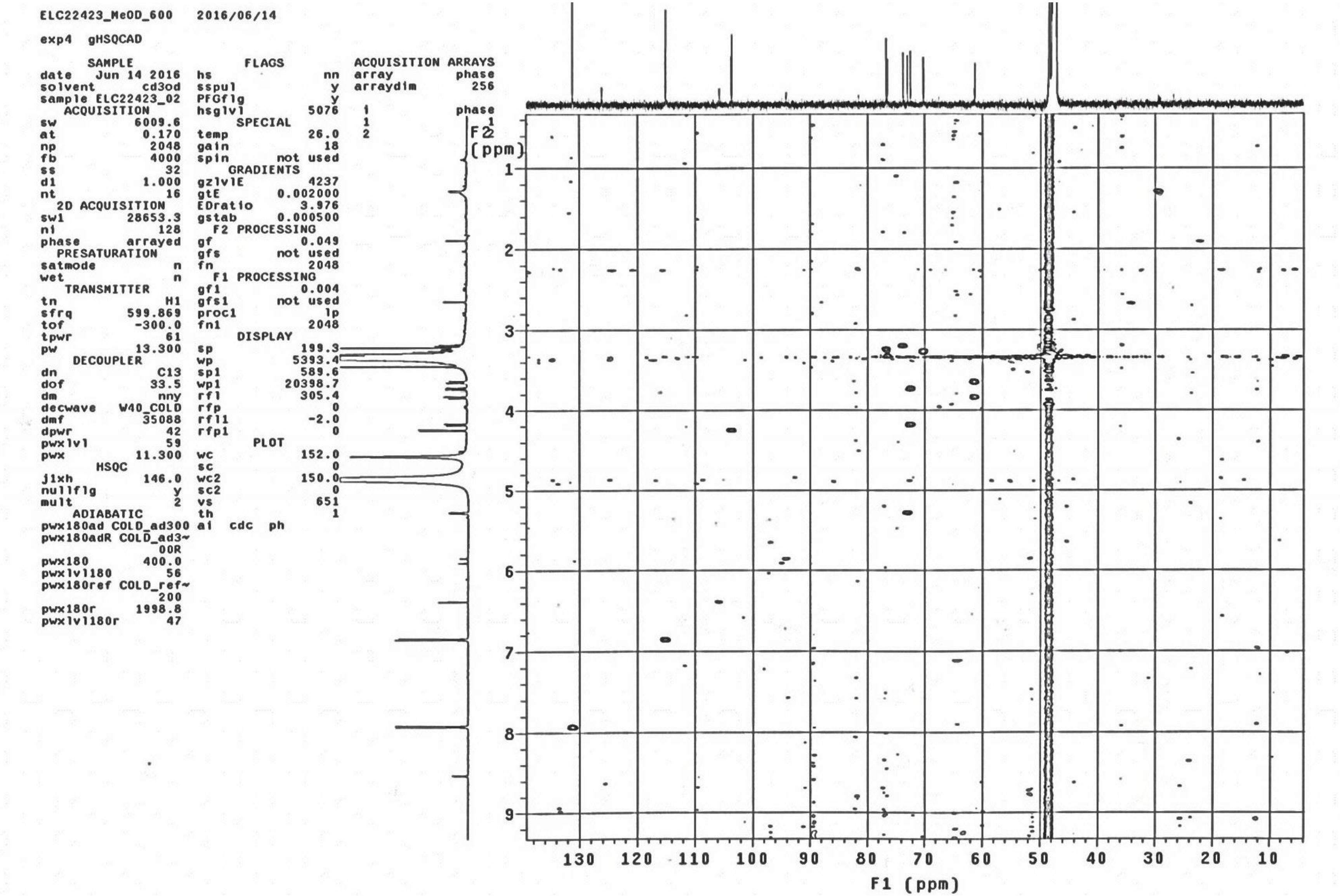

Figure S11. gHSQCAD spectrum of compound **11**, measured in MeOH-d_4

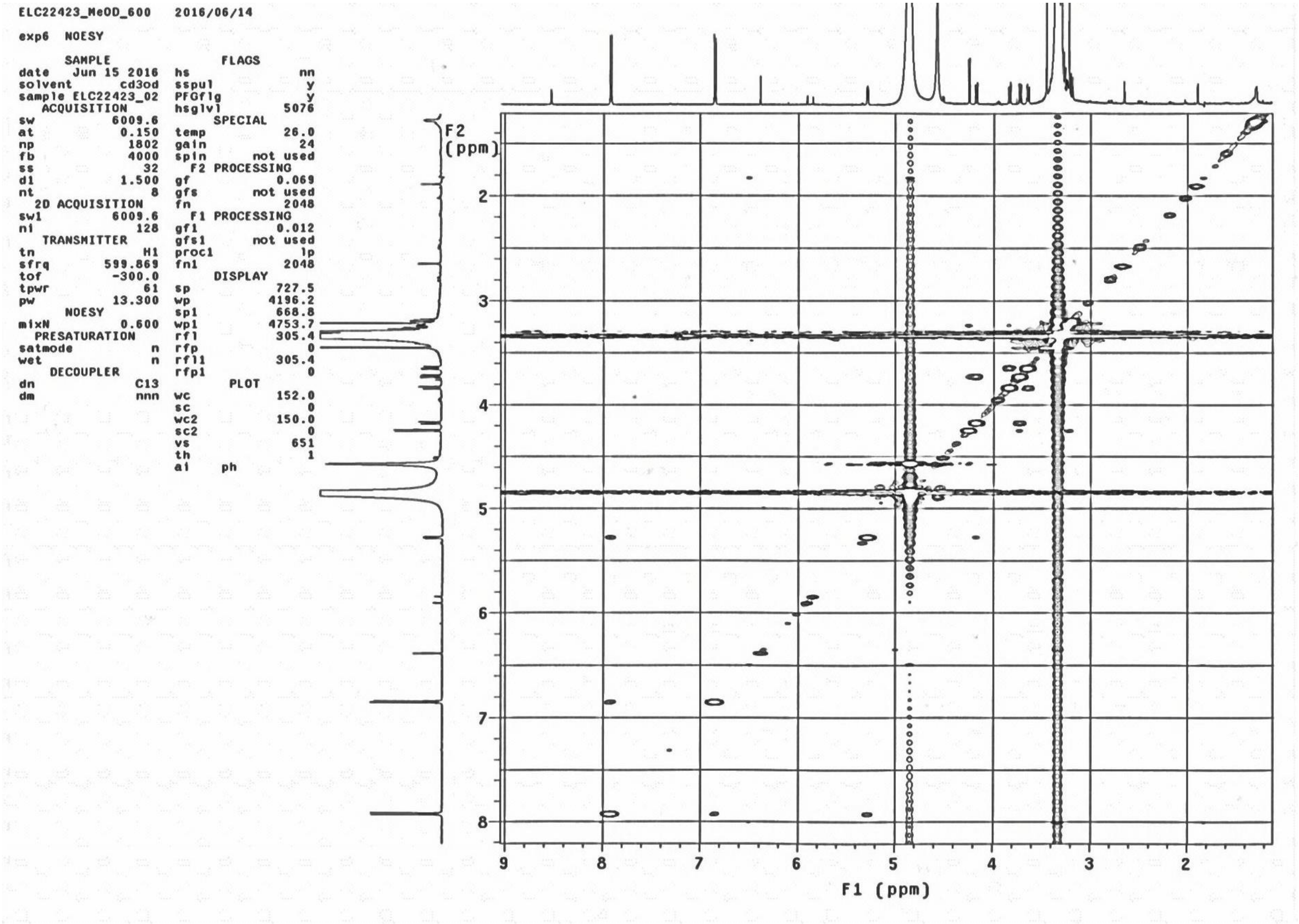

Figure S12. NOESY spectrum of compound **11**, measured in MeOH-d_4

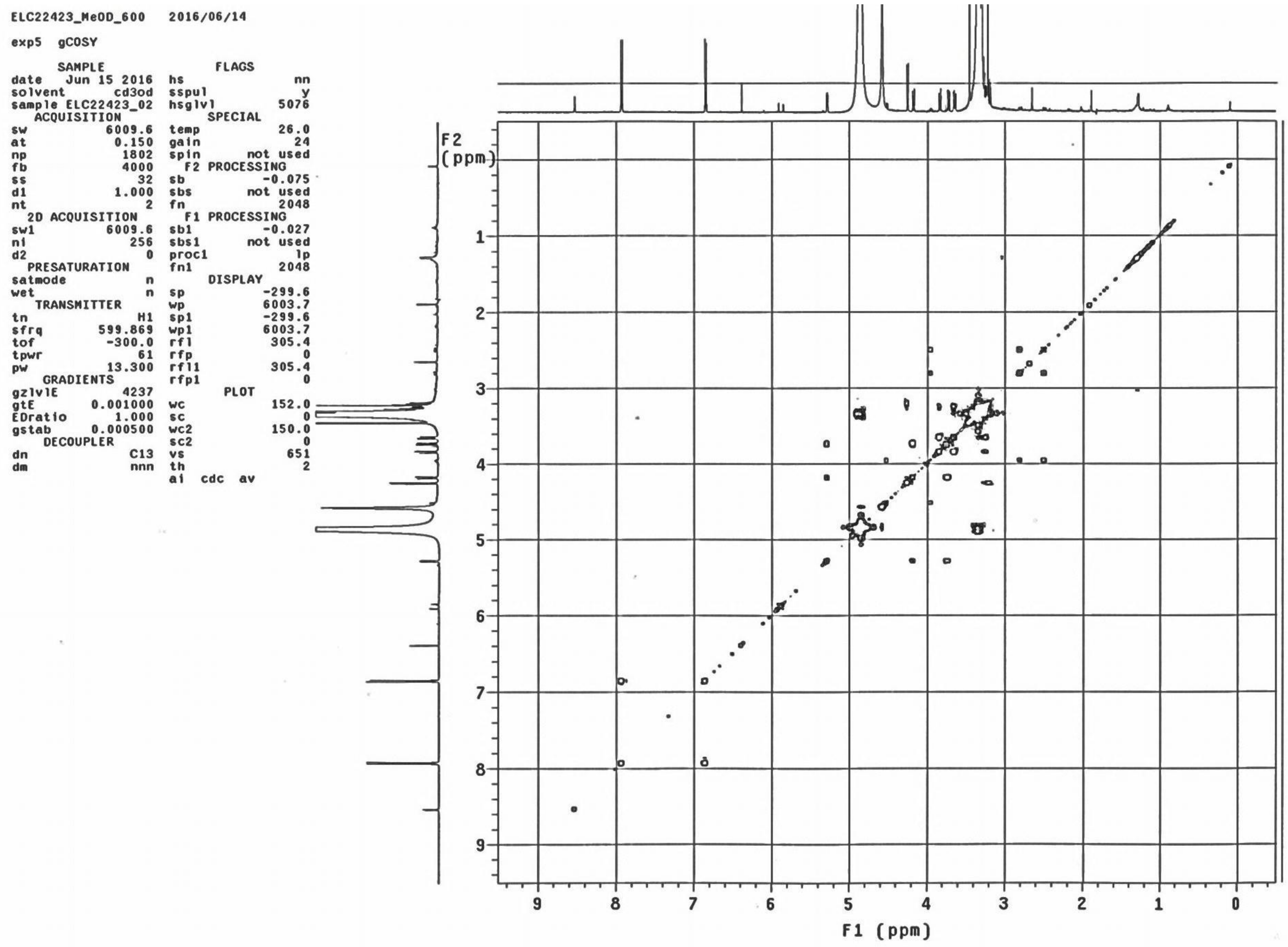

Figure S13. gCOSY spectrum of compound **11**, measured in MeOH-d_4

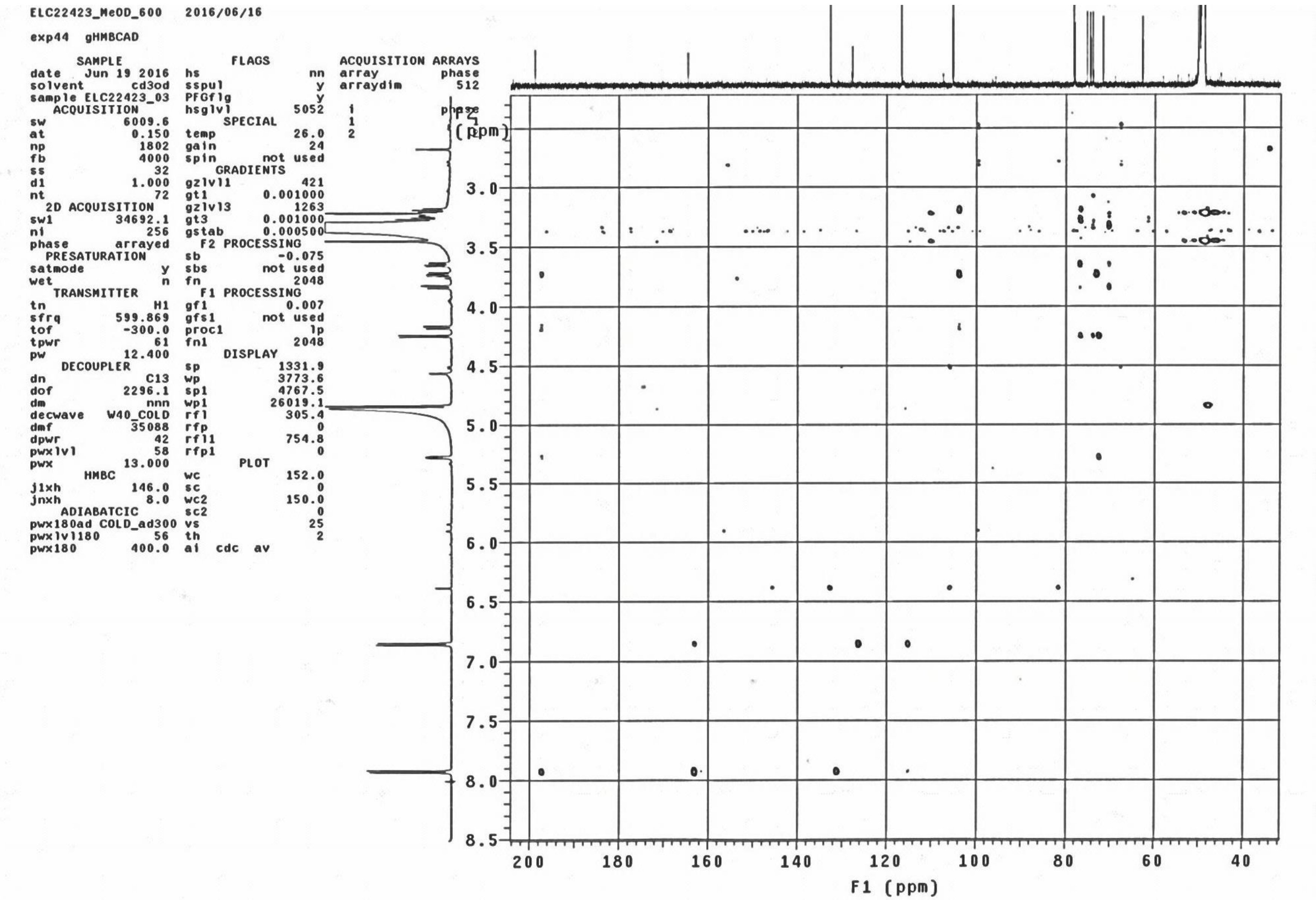

Figure S14. gHMBCAD spectrum of compound **11**, measured in MeOH-d_4

1.3. NMR spectrums of Compound 12, 1-*O*-(3-methyl)-butenoyl-myo-inositol: Figure S15 – Figure S21

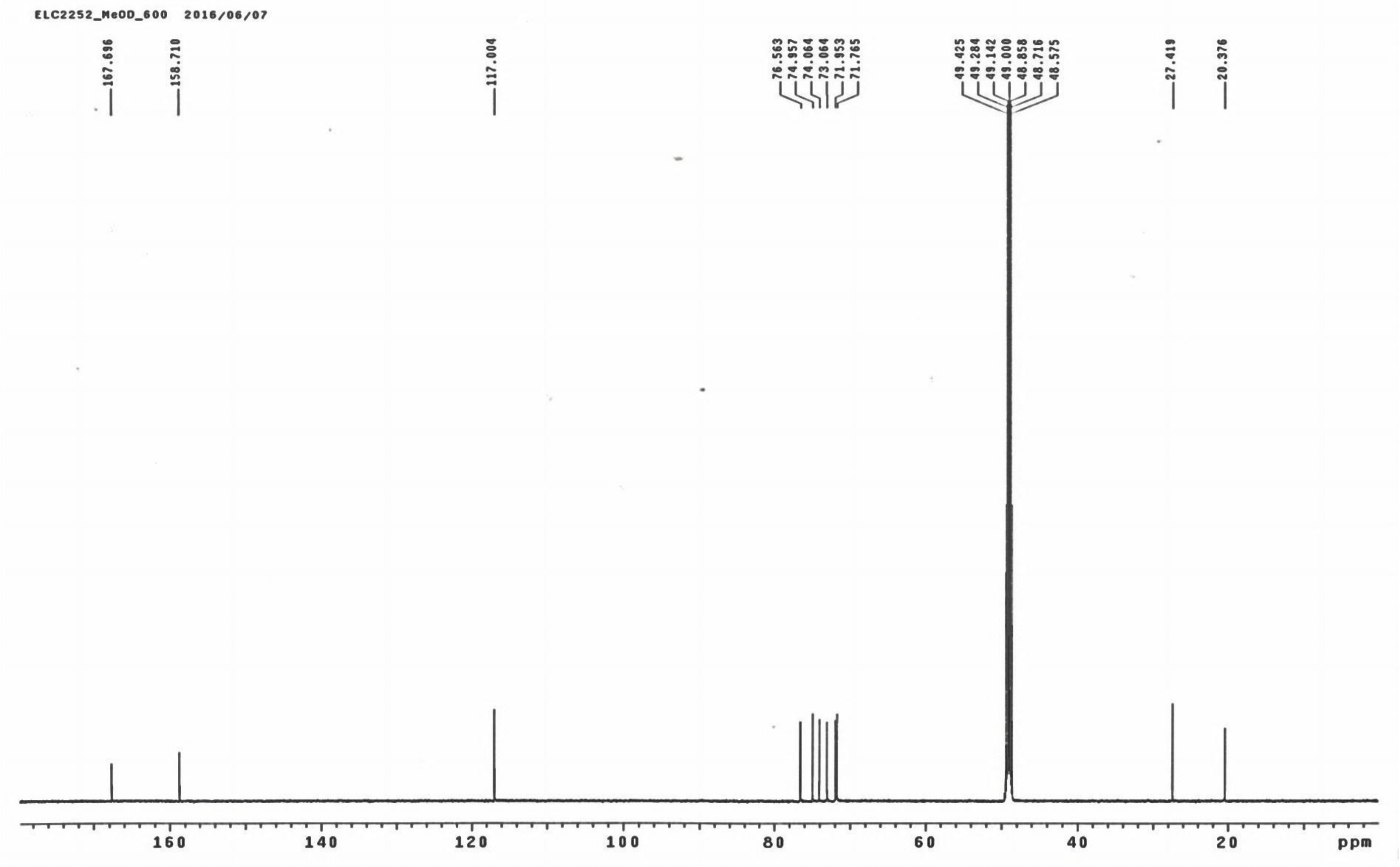

Figure S15. ^{13}C-NMR spectrum of compound **12**, measured in MeOH-d_4 at 150 MHz

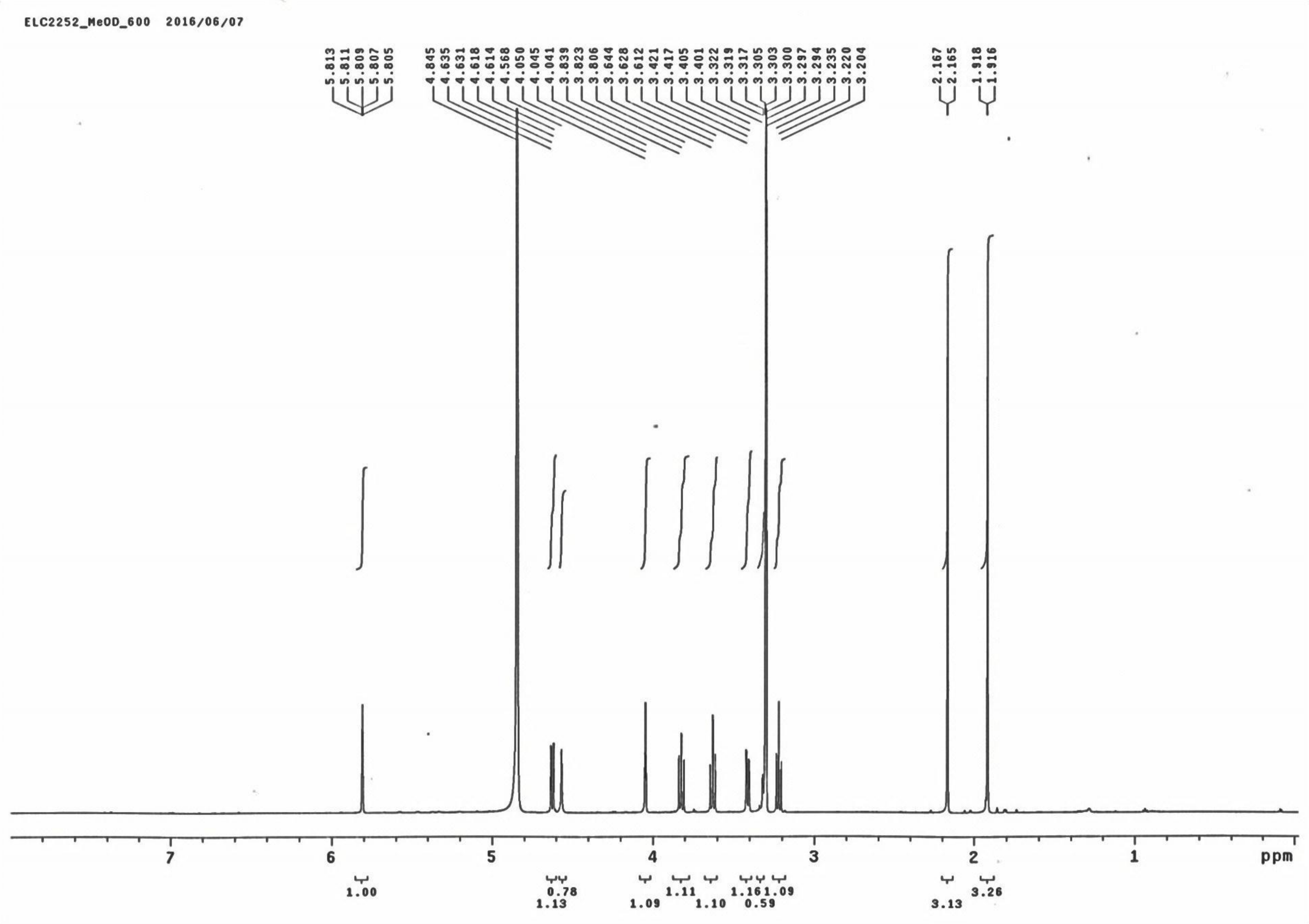

Figure S16. ^{1}H NMR spectrum of compound **12**, measured in MeOH-d_4 at 600 MHz

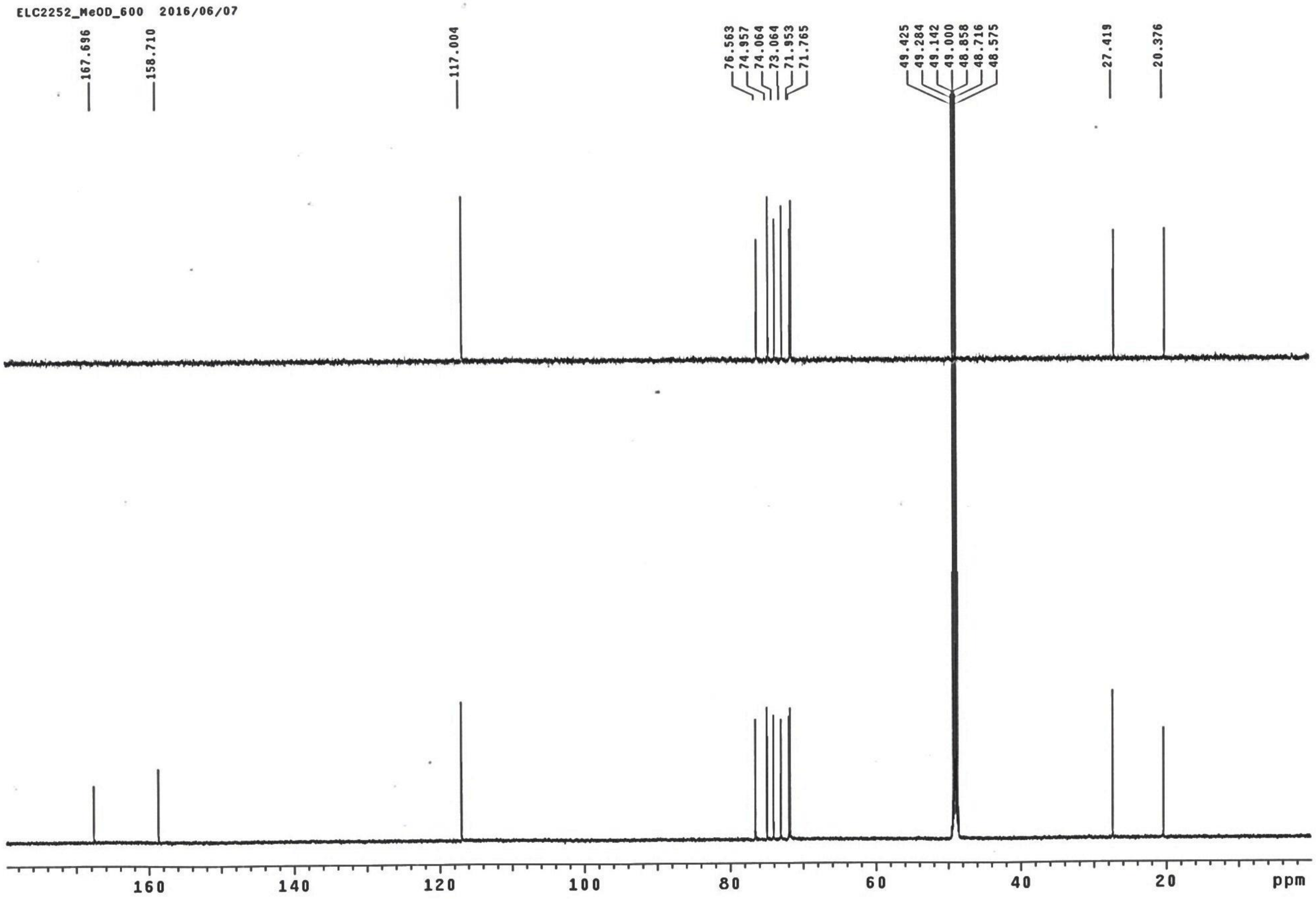

Figure S17. DEPT135 spectrum of compound **12**, measured in MeOH-d_4 at 150 MHz

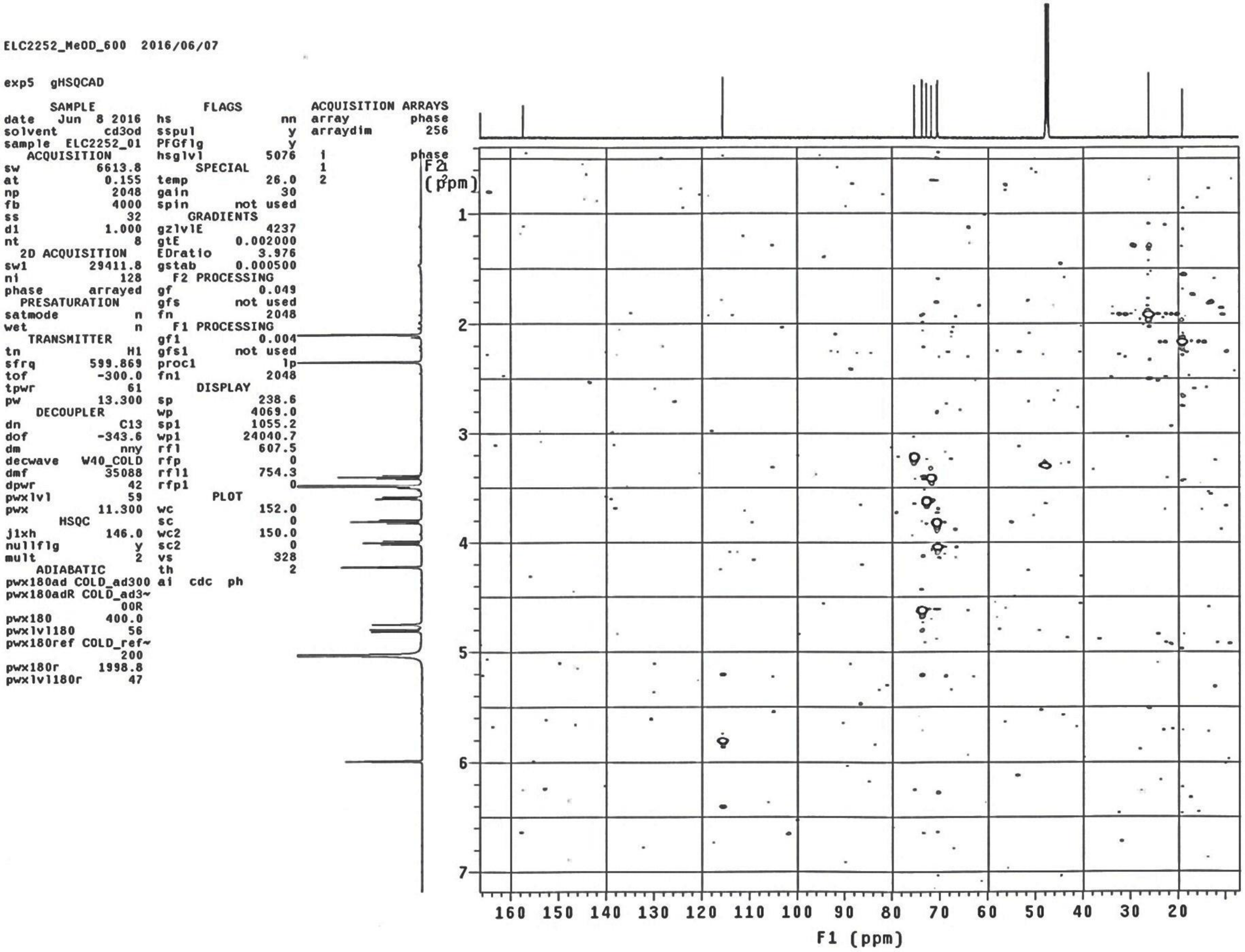

Figure S18. gHSQCAD spectrum of compound **12**, measured in MeOH-d_4

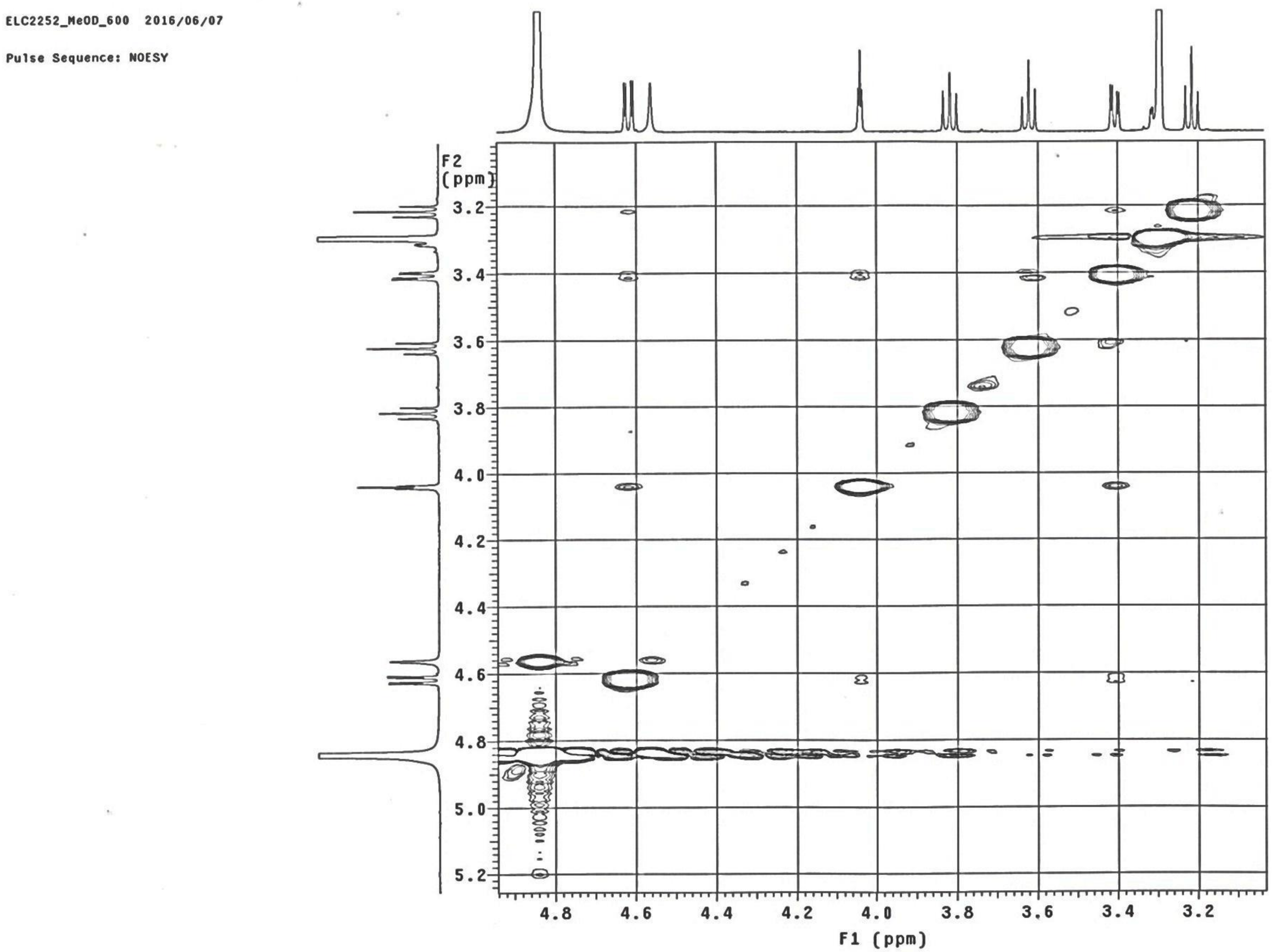

Figure S19. NOESY spectrum of compound **12**, measured in MeOH-d_4

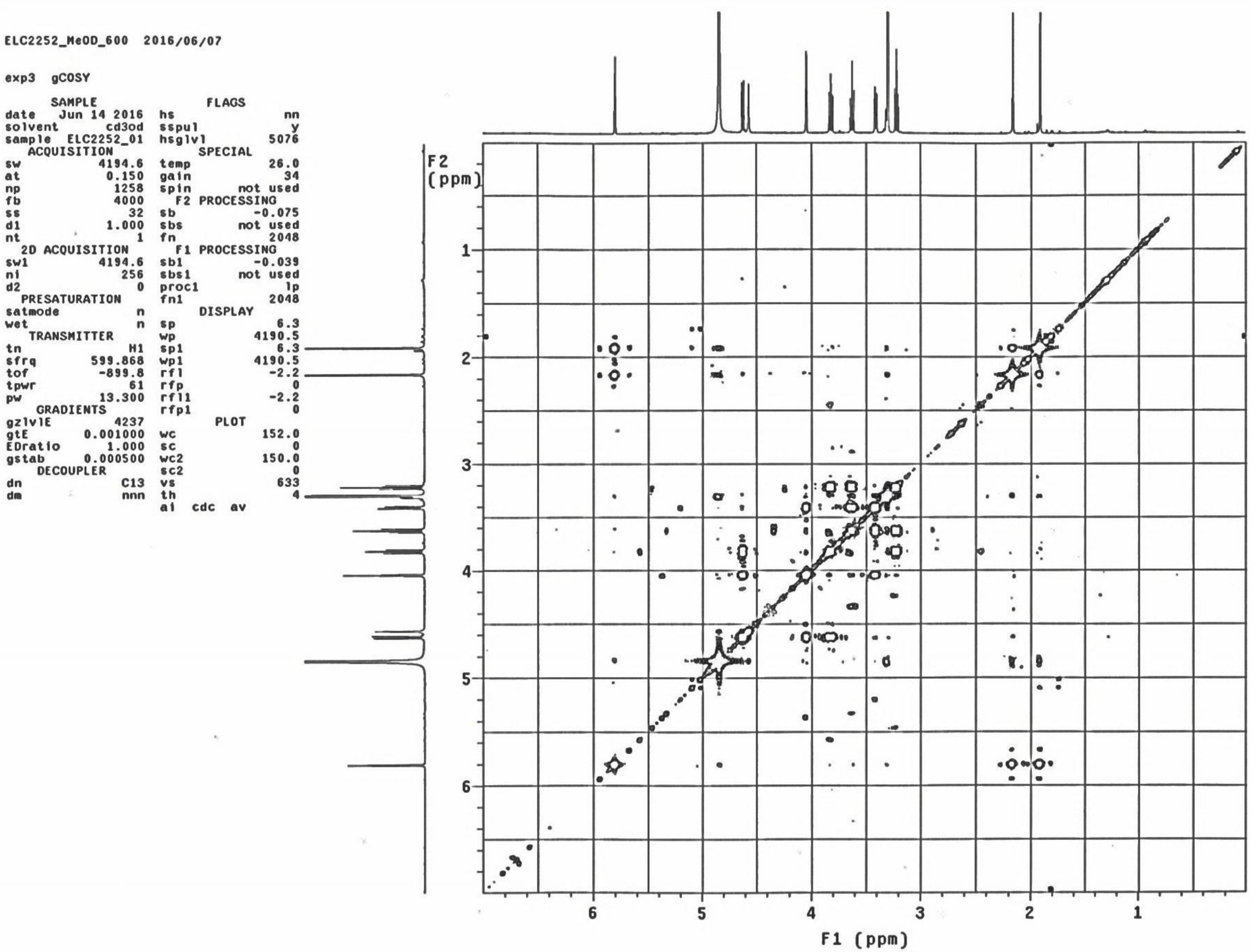

Figure S20. gCOSY spectrum of compound **12**, measured in MeOH-d_4

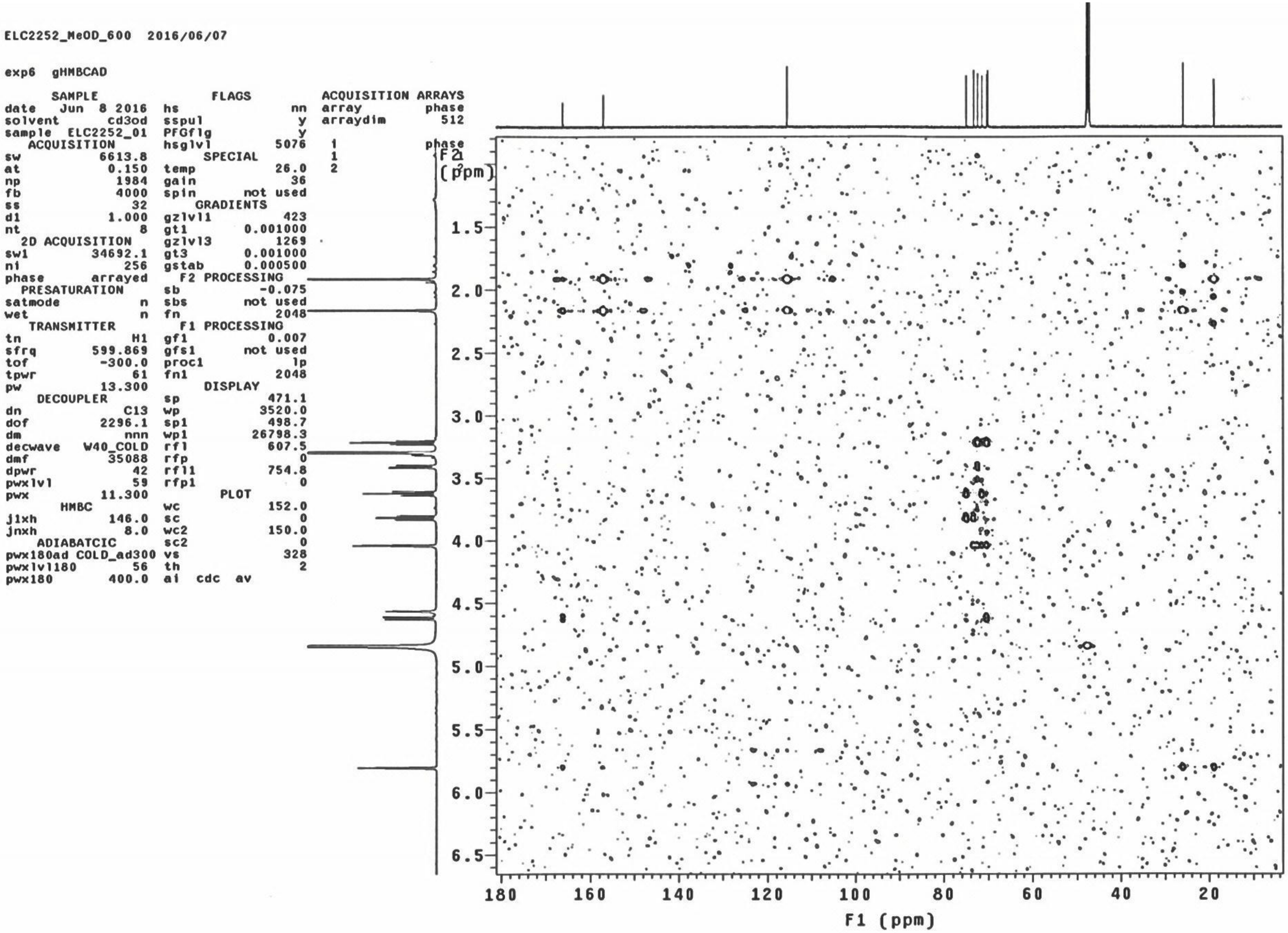

Figure S21. gHMBCAD spectrum of compound **12**, measured in MeOH-d_4

2. IC50 plots for all tested compounds: 1, 5, 10, 11, 12 14, 18, 19, and 20 (Figure S22 – Figure S30), and acarbose (Figure S31)

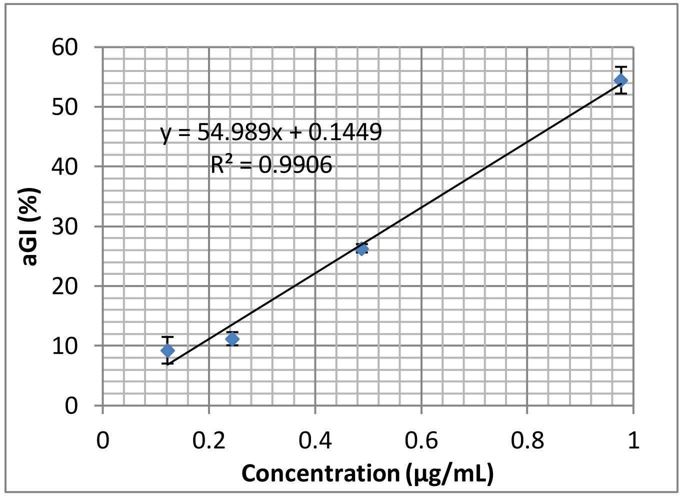

Figure S22. IC50 plots for compound 1: Walterolactone A/B β-D-pyranoglucoside

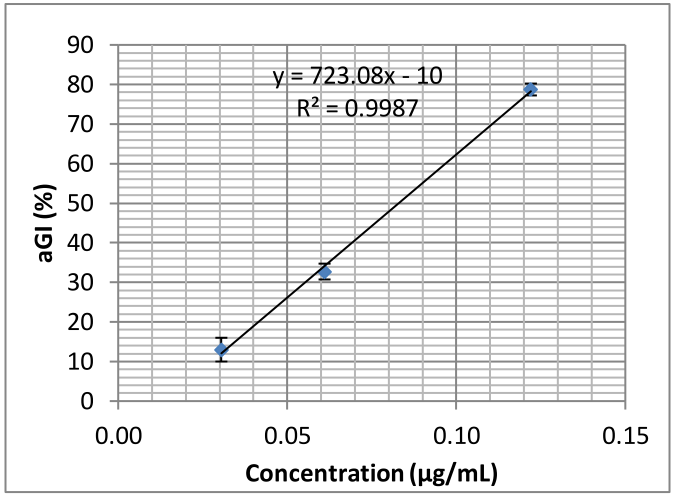

Figure S23. IC50 plots for compound 5: Condensed tannin-ELCTB-2.1.2

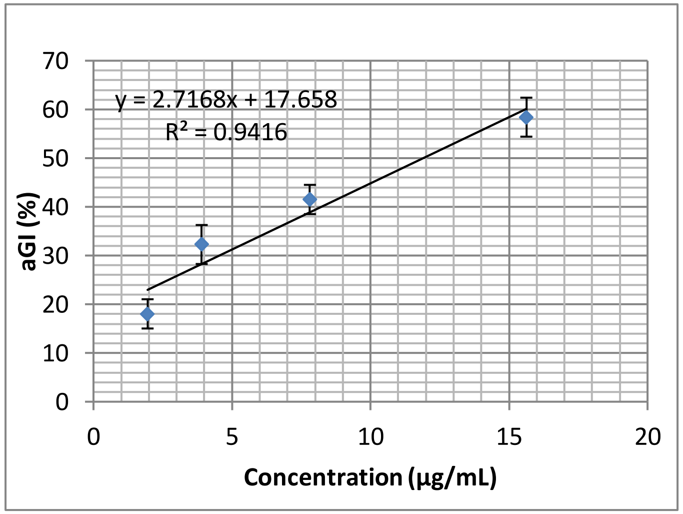

Figure S24. IC50 plots for compound 10: (−)-Gallocatechin

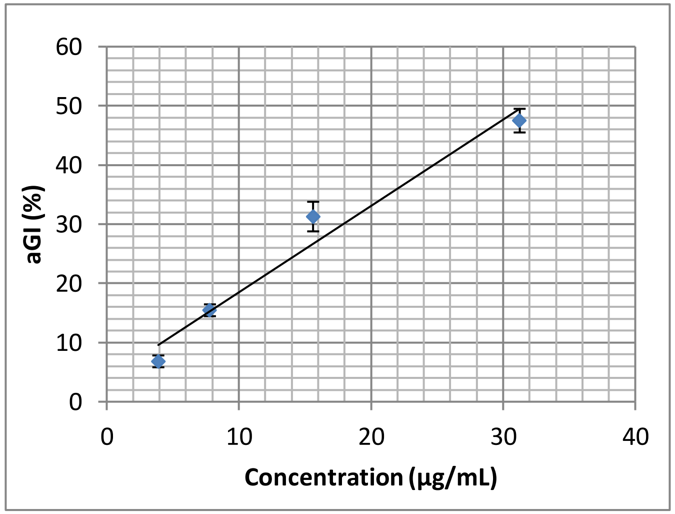

Figure S25. IC50 plots for compound 11: Schweinfurthinol 9-O-β-D-pyranoglucoside

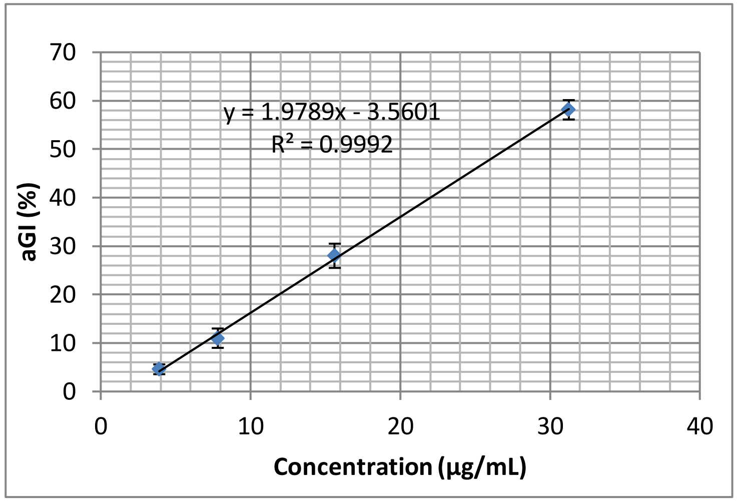

Figure S26. IC_{50} plots for compound 12: 1-O-(3-Methyl)-butenoyl-myo-inositol

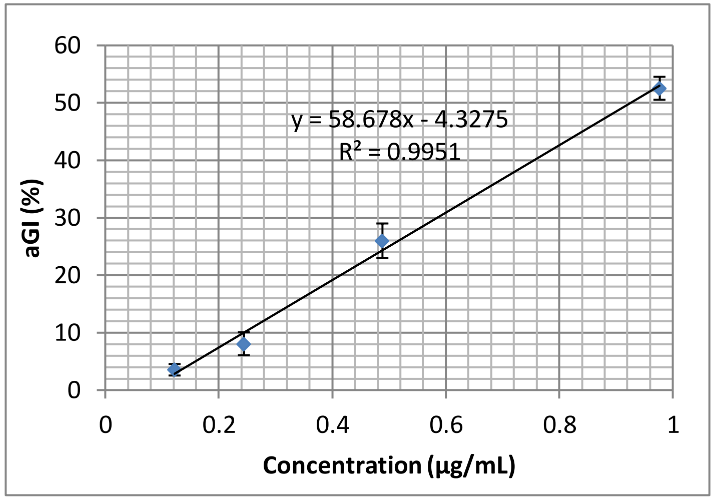

Figure S27. IC_{50} plots for compound 14: Leonuriside

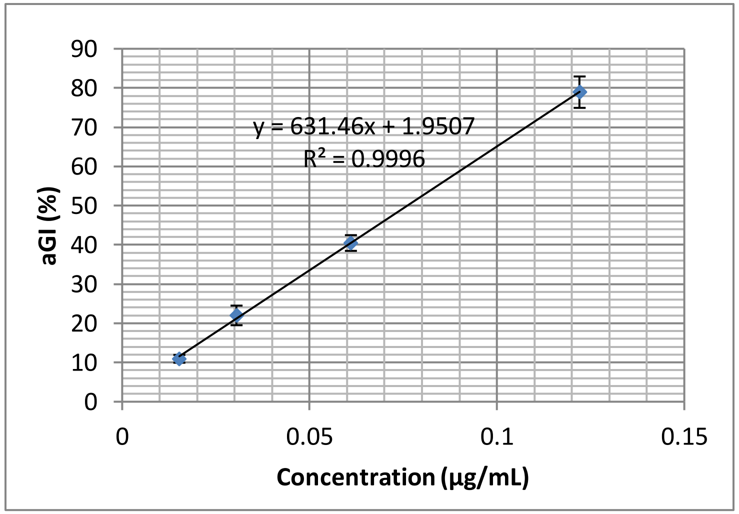

Figure S28. IC_{50} plots for compound 18: Condensed tannin-ELCTB-3.1

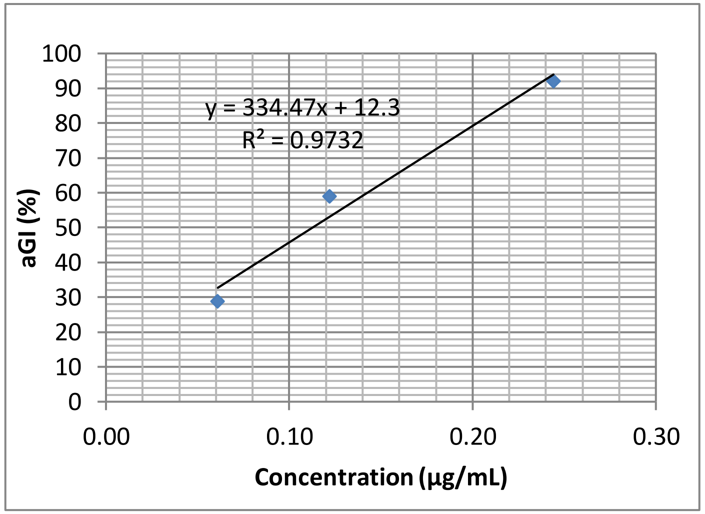

Figure S29. IC_{50} plots for compound 19: (+)-Catechin

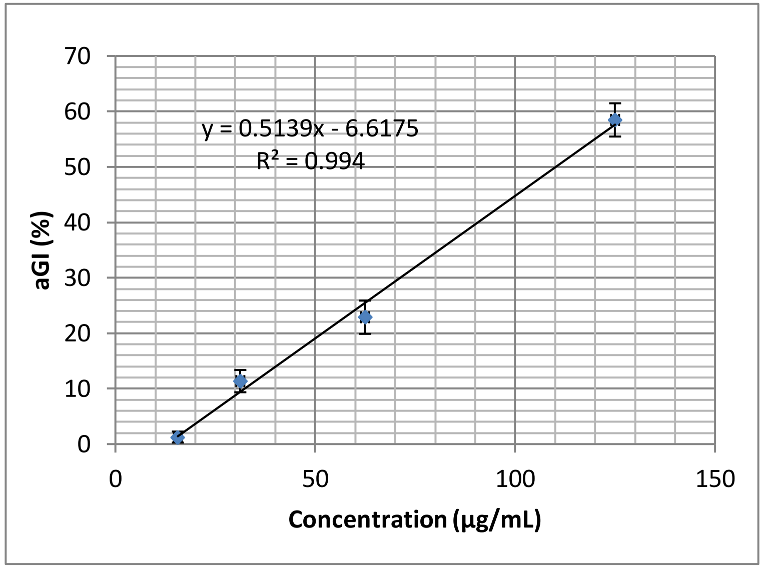

Figure S30. IC_{50} plots for compound 20: Methyl galloate

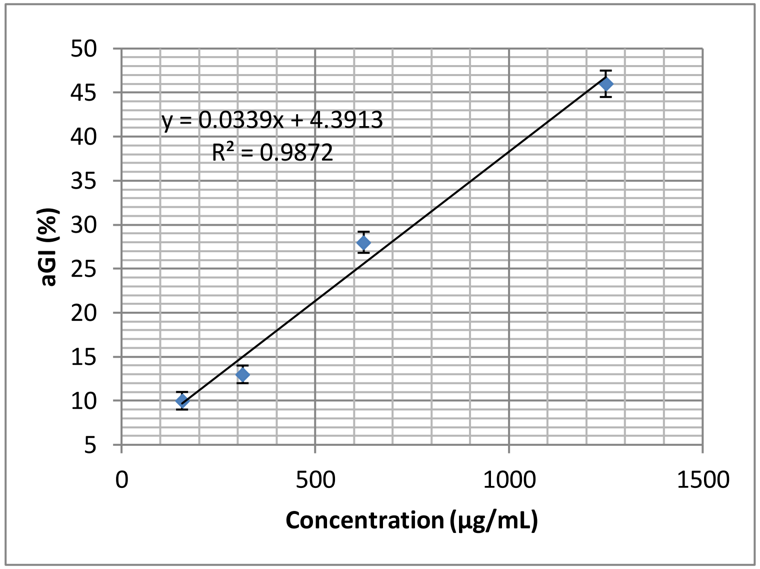

Figure S31. IC_{50} plots for acarbose

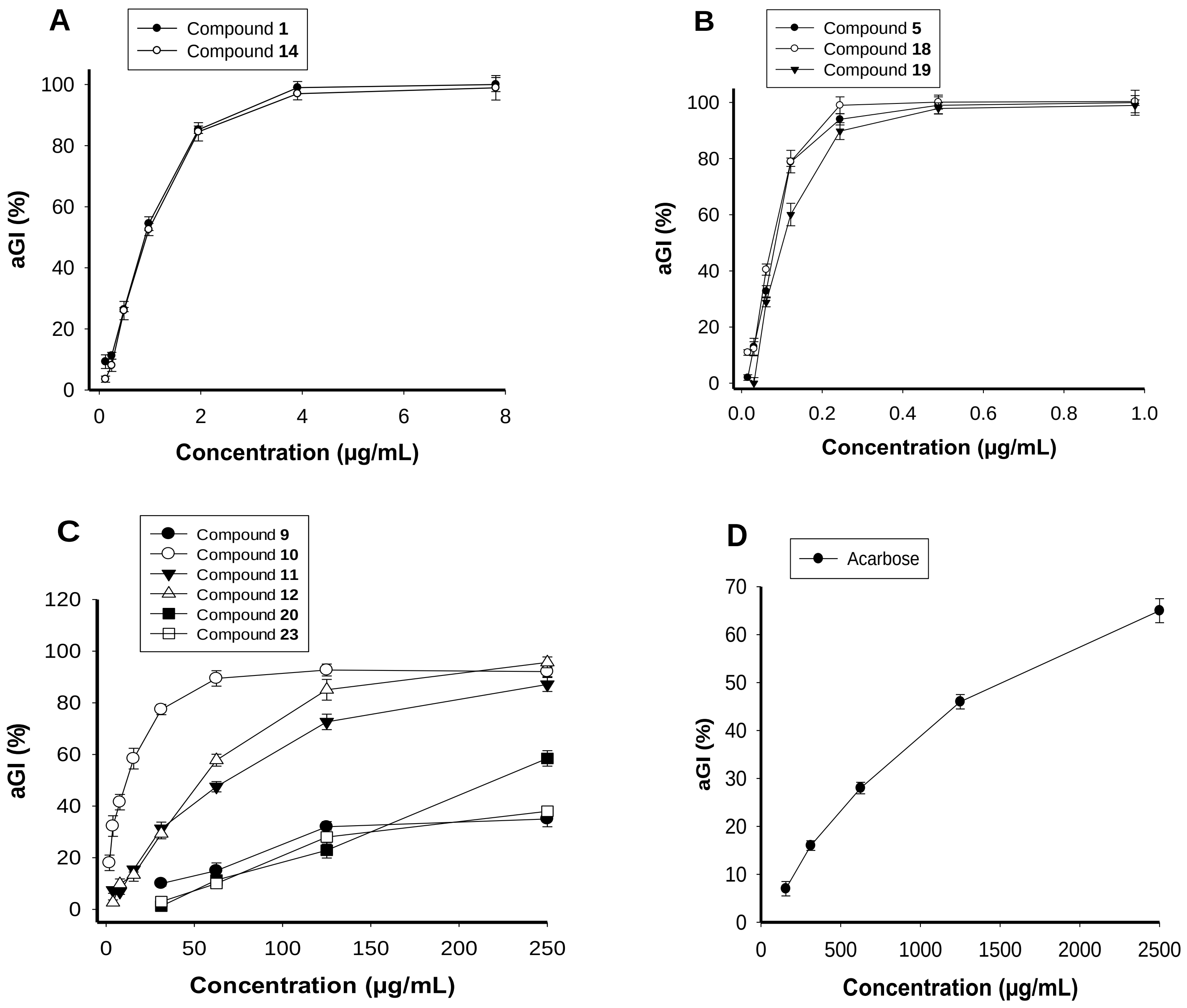

Figure S32. The corresponding % inhibition at each concentration of compounds **1** and **14** (**A**), compounds **5, 18** and **19** (**B**), compounds **9, 10, 11, 12, 20** and **23** (**C**), and acarbose (**D**).
